# Network Pharmacology and Transcriptomics Reveal the Mechanism of GuaLouQuMaiWan in Treatment of Type 2 Diabetes and Its Active Small Molecular Compound

**DOI:** 10.1155/2022/2736504

**Published:** 2022-10-06

**Authors:** Jiahao Feng, Yuheng Zhou, Li Liao, Liping Yu, Ping Yuan, Jun Zhang

**Affiliations:** ^1^Scientific Research Center, The Seventh Affiliated Hospital of Sun Yat-sen University, Shenzhen, China 518000; ^2^Department of Thoracic Oncology, Sun Yat-sen University Cancer Center, Guangzhou, China 510632; ^3^Chongqing Jiangjin District Hospital of Chinese Medicine, Chongqing, China 404100; ^4^Shenzhen Hospital of Traditional Chinese Medicine, Shenzhen, China 518000; ^5^Tongren Hospital Shanghai Jiao Tong University, Shanghai, China 200000; ^6^School of Traditional Medicine, Jinan University, Guangzhou, China 510632

## Abstract

The main pathophysiological abnormalities in type 2 diabetes (T2D) include pancreatic *β*-cell dysfunction and insulin resistance. Due to hyperglycemia, patients receive long-term treatment. However, side effects and drug tolerance usually lead to treatment failure. GuaLouQuMaiWan (GLQMW), a common traditional Chinese medicine (TCM) prescription, has positive effects on controlling blood sugar and improving quality of life, but the mechanism is still unclear. To decipher their molecular mechanisms, we used a novel computational systems pharmacology-based approach consisting of bioinformatics analysis, network pharmacology, and drug similarity comparison. We divided the participants into nondisease (ND), impaired glucose tolerance (IGT), and type 2 diabetes groups according to the WHO's recommendations for diabetes. By analyzing the gene expression profile of the ND-IGT-T2D (ND to IGT to T2D) process, we found that the function of downregulated genes in the whole process was mainly related to insulin secretion, while the upregulated genes were related to inflammation. Furthermore, other genes in the ND-IGT (ND to IGT) process are mainly related to inflammation and lipid metabolic disorders. We speculate that 17 genes with a consistent trend may play a key role in the process of ND-IGT-T2D. We further performed target prediction for 50 compounds in GLQMW that met the screening criteria and intersected the differentially expressed genes of the T2D process with the compounds of GLQMW; a total of 18 proteins proved potential targets for GLQMW. Among these, RBP4 is considerably related to insulin resistance. GO/KEGG enrichment analyses of the target genes of GLQMW showed enrichment in inflammation- and T2D therapy-related pathways. Based on the RDKit tool and the DrugBank database, we speculate that (-)-taxifolin, dialoside A_qt, spinasterol, isofucosterol, and 11,14-eicosadienoic acid can be used as potential drugs for T2D via molecular docking and drug similarity comparison.

## 1. Background

Type II diabetes (T2D) is a chronic disease characterized primarily by abnormally high blood sugar levels. The number of patients, a high proportion of whom are young adults, suffering from T2D is increasing annually [1]. The World Health Organization (WHO) recommends that T2D be divided into two pathological states, impaired glucose tolerance (IGT) and T2D [2, 3], upon which doctors can adjust treatment strategies. The pathophysiological characteristics of T2D include insulin resistance and insulin deficiency [4]. Obesity due to disordered glucose and lipid metabolism is a common cause of T2D. Insulin deficiency is mainly related to glucose toxicity [5] and lipotoxicity [6], which result in pancreatic *β*-cell dysfunction. After an irregular diet, an imperceptible and cumulative increase in fasting and 2 h postprandial blood glucose levels occurs [5], which is known as glucotoxicity. Glucose toxicity can cause pancreatic *β*-cell dysfunction and senescence. Moreover, compared with the loss of *β*-cells, supplementation through the differentiation of islet stem/progenitor cells [7] and self-replication of pancreatic *β*-cells [8] is insignificant, which leads to insufficient insulin secretion and abnormal blood sugar levels. In a cohort study of T2D, researchers measured the quality of various parts of the pancreatic tissue and found that the *β*-cell content of T2D and IGT patients showed a downward trend [9]. Insulin resistance refers to a phenomenon in which the biological effects of insulin in muscle tissue and the liver, such as promoting the production of muscle glycogen and liver glycogen, are reduced [10]. Insulin can regulate blood glucose homeostasis by stimulating the phosphatidylinositol 3-kinase (PI3K)-independent pathway through binding and activating membrane-localized receptors using tyrosine kinase activity [11]. Similarly, insulin has a regulatory effect on lipids and glycogen, including lipid conversion, glycogen decomposition and synthesis, and glucose transporters activity increase on the cell surface, affecting mRNA synthesis, cell proliferation, and survival [12]. In insulin resistance, tyrosine kinase receptor (the main insulin receptor) cannot adequately activate downstream reactions because the expression of protein-tyrosine phosphatase 1B (PTP1B) tends to be upregulated, exerting a dephosphorylation effect that counteracts the biological effects of insulin [13]. Moreover, insulin resistance can cause chronic inflammation and a decrease in cell surface receptors. First, members of the cytokine signaling inhibitor protein family activate degradation of the cell membrane surface insulin receptor substrate through the ubiquitin-proteasome pathway [14]. Second, the massive release of free fatty acids and cellular inflammatory factors, such as tumor necrosis factor (TNF) and interleukin-6 (IL6), also has a negative impact on the biological effects of insulin [15, 16]. These factors accelerate T2D progression.

In China, GuaLouQuMaiWan (GLQMW), a traditional prescription, synergizes with antidiabetic drugs in T2D treatment by reducing drug resistance [17] and stabilizing blood sugar [18]. GLQMW, which is composed of *Trichosanthis* Radix (THF), *Aconiti Lateralis Radix Praeparata* (FZ), *Poria Cocos* (Schw.) *Wolf.* (FL), *Dianthi Herba* (QM), and *Rhizoma Dioscoreae* (SY), can invigorate the spleen and kidney and eliminate dampness and diuresis, according to the principles of TCM for T2D. SY and FL proved to be effective in intervening blood glucose and blood lipid levels in animal models [19, 20], and QM has an anti-inflammatory effect [21]. Although recent research has shown that some components of GLQMW are beneficial to T2D, there has been no research to interpret the specific molecular mechanism of GLQMW intervention in the progression of T2D.

T2D is a nonmutated disease, hence, its progression is laboriously illustrated through the change of a single gene. However, the changes in gene expression profiles during disease progression can be identified and visualized by transcriptome sequencing analysis [22], providing insight on potential therapeutic targets. TCM is a multitarget and multipath process for the treatment of diseases, so we need to separate and analyze natural compounds in herbs that meet potential drug standards. We can summarize the effective compounds of GLQMW in the Traditional Chinese Medicine Systems Pharmacology Database and Analysis Platform (TCMSP) as it collects the herbs contained in the Chinese Pharmacopoeia and the basic physical and chemical properties of their related compounds [23]. However, we must choose the PharmMapper server, a tool based on the pharmacophore mapping algorithm [24], to predict potential targets of the compound since TCMSP only includes the confirmed information for the herbal compound. The PharmMapper server is designed to identify potential target candidates for the given probe small molecules (drugs, natural products, or other newly discovered compounds with binding targets unidentified) using a pharmacophore mapping approach in the pharmacophore database (PharmTargetDB) and lists the annotation and classification of the predicted target [25]. The principle of drug similarity means that the structure of a compound determines its basic properties, therefore, the more similar the structure of a compound, the more similar the basic properties, such as physical and chemical properties and physiological metabolism [26]. This principle helps us to further decipher the potential pharmacological activities of compounds that are not included in the PharmMapper server. Furthermore, RDKit can help us compare the structure of small molecule compounds, especially in the field of pharmacological identification of TCM [27]. Based on the Python environment, RDKit, an open-source toolkit suitable for chemical informatics [27], can convert 2D/3D to 3D/2D compound structures, generate compound fingerprints, and calculate the structure of compound similarity via machine learning methods [28]. Following RDKit processing, we deduced that GLQMW contains small-molecule compounds that interfere with the process of T2D progression. Finally, molecular docking and LIGPLOT can predict binding poses and affinities through the interactions between receptors and drug molecular ligands, which involve spatial matching and energy matching between molecules [29]. These methods are powerful tools for pharmacological mechanistic and drug research and development. Therefore, transcriptomic analysis, network pharmacology, and molecular docking were used to explore the pharmacological targets and mechanisms of GLQMW against T2D.

## 2. Materials and Methods

**Figure 1:**
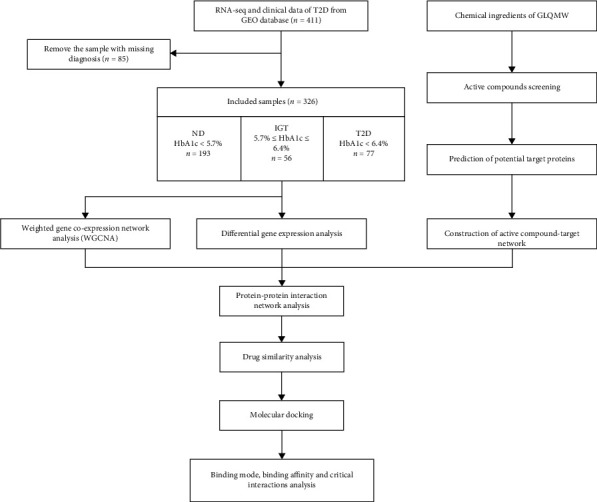
Experimental design for elucidating the mechanisms of action of GLQMW in the treatment of T2D.

The overall process of the research is shown in Figure 1.

### 2.1. Raw Data

RNA-seq data of T2D (including 411 islet tissue samples) and the corresponding clinical data were downloaded from GEO dataset. By filtering out the sample missing diagnosis, the expression data for 326 islet tissue were kept for downstream analysis. Four cohort used in this study as validation datasets (GSE38642, GSE50397, GSE76894, and GSE76895) were downloaded from the GEO database (https://www.ncbi.nlm.nih.gov/geo). Compounds of GLQMW and its' target proteins origin from TCMSP (https://tcmspw.com/) and PharmMapper server, respectively.

### 2.2. Type 2 Diabetes Process Classification

Using the diagnostic criteria (ND < 5.7%, IGT5.7%-6.4%, T2D : >6.4%) which is recommended by WHO, we distinguished the T2D process of diabetic patients via glycosylated hemoglobin (HbA1c), and divided the samples into 3 groups (including 193 ND, 56 IGT, and 77 T2D islet tissue samples).

### 2.3. Differential Gene Expression Analysis

Differential expression analysis was conducted using the R package “limma”. The screening conditions for the differential genes were |log2FoldChange| > 0.3, *p*.adj < 0.05. Heatmaps of differential genes were drawn using the R package “pheatmap”. For process-specific genes, only genes with significant differences and consistent trend in expression (|log2FC| > 0.3, *p*.adj < 0.05) in all three possible comparisons were considered T2D-specific genes.

### 2.4. Weighted Gene Coexpression Network Analysis (WNGCA) and Identification of Clinically Significant Modules

We use the R package “WGCNA” to construct a gene-weighted coexpression network, when R package “limma” has processed the gene expression data of removing the batches. First, compared to the Pearson method, we chose the biweight midcorrelation method to construct the adjacency matrix to describe the correlation strength between nodes. Subsequently, choosing the soft threshold *β* = 4 (scale free *R*^2^ = 0.85), we convert the adjacency matrix to a topological overlap matrix (TOM), and set the type of TOM to a signed network. Next, we perform hierarchical clustering to identify modules which contains at least 30 genes (minModuleSize = 30), calculated feature genes, hierarchically clustered the modules, and merged similar modules.

The module feature gene (ME) is the first principal component of the module and represents the expression pattern of the module in each sample. The degree of module membership (MM) refers to the correlation coefficient between genes and the characteristic genes of the module, which is used to describe the reliability of the gene belonging to the module. Based on ME and MM, we calculated the correlation between modules and clinical data to determine important clinical modules. Finally, we selected the modules whose expression changes were in line with the process of ND-IGT-T2D, and extracted the hub gene and core genes which were further analyzed.

### 2.5. GO and KEGG Enrichment Analysis

GO and KEGG enrichment analyses were performed with the aid of R packages “clusterProfiler,” “enrichplot,” and “ggplot2.” Only terms with both *p* and *q* value of < 0.05 were considered significantly enriched. For T2D, GO and KEGG enrichment analyses were based on upregulated and downregulated expressed genes. For GLQMW, we used “http://org.hs.eg.db/” to convert the target protein into a gene, and then performed GO and KEGG enrichment analysis.

### 2.6. Active Compounds Screening

All of the chemical ingredients of GLQMW were obtained from TCMSP. The active compounds of GLQMW were mainly filtered with oral bioavailability (OB), drug-likeness (DL), and Caco-2 permeability (Caco-2), while those compounds in GLQMW with OB ≥ 30%, DL ≥ 0.18, and Caco − 2 ≥ −0.4 were preserved [30]. But, we selected 5 compounds with reports of pharmacological activity to join the results, and those compounds' OB and DL meet the threshold but Caco-2 does not.

### 2.7. Network Pharmacology Analysis

Download the 3D structure of the GLQMW compound from TCMSP. The PharmMapper server recognizes the pharmacophore of the compound, and uses the pharmacophore as the recognition group of given probe molecule to predict potential target proteins. PharmMapper server output results, including target name and protein number, among which *z*-score is an important basis for target fit. We choose 1.0 as the threshold, and extract all protein targets greater than the threshold for subsequent analysis. Based on cytoscape, we construct an active compound-target network, and select compounds with a larger number of nodes for key analysis.

### 2.8. Drug Similarity Analysis

Download the “full database” document from the DrugBank database (https://go.drugbank.com/) [31], which contains all the drug details. In the process of processing the document, select the drug information whose type is “small molecule,” and remove the drug whose category is protein, due to focusing on small molecule compounds in GLQMW.

RDkit generates compound descriptors and compound fingerprints, and calculates the similarity of compound structures, based on the 2D and 3D structure of compounds.

Our study compares active compounds in GLQMW and small molecule drugs in the DrugBank database, and outputs information on the top 10 drugs with drug similarity.

### 2.9. Molecular Docking and LIGPLOT

The 3D structure of protein: RBP4 (PDB ID:2WR6, Uniprot:P02753), PLAT (PDB ID:1A5H, Uniprot:P00750), MET (PDB ID:2ZGH, Uniprot:P51124), KIT (PDB ID:4U0i, Uniprot:P10721), C1S (PDB ID:5UBM, Uniprot:P09871), and HSD11B (PDB ID:2RBE, Uniprot:P28845) were downloaded from the protein data bank (PDB) database (https://www.rcsb.org/). The ligand and water macromolecule in these targets were removed, and the hydrogen atoms were added with pymol2.3. The targets were set to rigid and saved as pdbqt file format by AutoDock Tools 1.5.6. Finally, molecular docking was performed using Vina. Based on the top one minimal binding energy of each target, compounds in GLQMW were selected for further analysis of their binding mode, binding affinity, and critical interactions using PyMOL2.3 and LIGPLOT2.2.

## 3. Results

### 3.1. Remove Different Batch Effect

This study included high-throughput gene expression profiling data of 326 pancreatic islet tissue samples from 4 datasets. Since the data comes from different sequencing platforms, the data has a relatively obvious batch effect, which will adversely affect the results of subsequent analysis. Therefore, we eliminate the batch effect on the combined data, and the results are shown in Supplementary Figure 1.

### 3.2. Identification of Type 2 Diabetes Progress Important DEGs by Transcriptome Analysis

**Figure 2:**
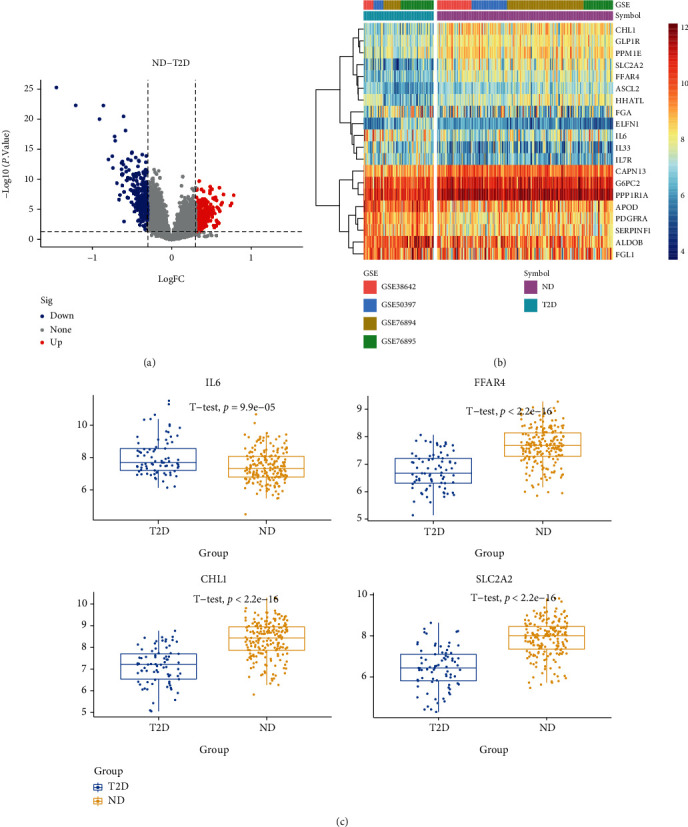
Volcano plots, heatmaps, and gene expression profiles of DEGs in ND-T2D. (a) Number and distribution of up and downregulated genes. (b) Heatmap for DEGs generated by comparison in ND and T2D. Row is the gene, and column name is the samples, which is not shown in plot. DEGs were determined by Wilcoxon rank sum test with *q* < 0.05 and log2FC > 0.3 as the significance threshold. (c) Expression levels of some DEGs in ND and T2D.

Using ND as the control, 520 differential genes were identified in the ND-T2D group, of which 259 were upregulated and 261 were downregulated. Using ND as the control, 58 upregulated genes and 42 downregulated genes were identified in the IGT group. In the comparison between the IGT-T2D groups and IGT as the control, there were 156 upregulated genes and 70 downregulated genes in T2D.  Figure 2 shows DEGs expression in ND-T2D, Supplementary Figures 2 and 3 show DEGs expression in ND-IGT and IGT-T2D, respectively.

In ND-T2D, the most significantly upregulated and downregulated genes were APOD (logFC = 0.77, *p*.adj < 0.05) and SLC2A2 (logFC = −1.45, *p*.adj < 0.05), respectively. In the ND-IGT, the most significantly upregulated and downregulated genes were PTGS2 (logFC = 0.58, *p*.adj < 0.05) and TMED6 (logFC = −0.68, *p*.adj < 0.05), respectively. In IGT-T2D, the most significantly upregulated and downregulated genes were CEACAM7 (logFC = 0.91, *p*.adj < 0.05) and SLC2A2 (logFC = −0.99, *p*.adj < 0.05), respectively.

**Figure 3:**
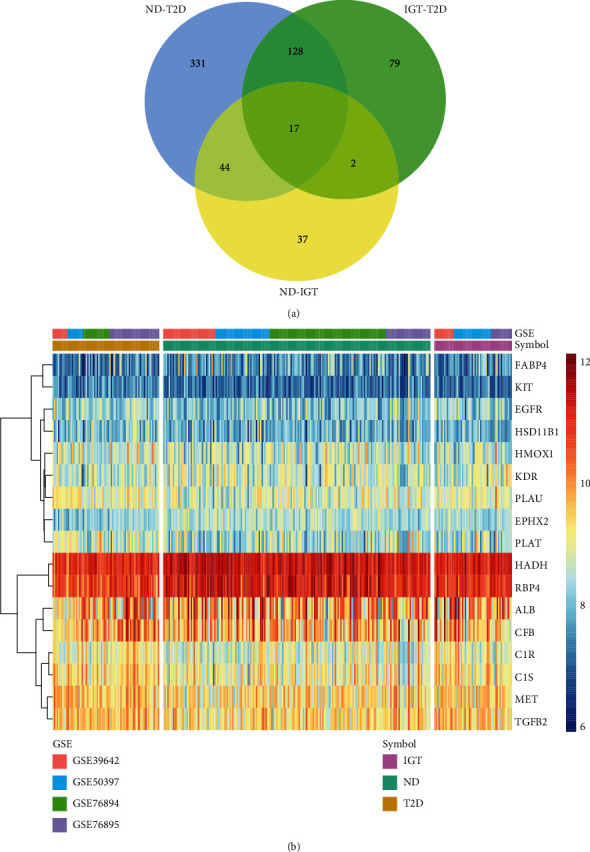
Venn diagrams and heatmaps of intersecting genes in ND-IGT-T2D. (a) Venn plots showing common upregulated and downregulated DEGs shared by ND-IGT, IGT-T2D, and ND-T2D. (b) The expression level of common up and downregulated DEGs in ND, IGT, and T2D.

We found that the number of DEGs decreased among the three groups of ND-T2D (520), IGT-T2D (226), and ND-IGT (100), Figure 3. This phenomenon coincides with the clinical observation that the states of ND and IGT are somewhat similar. However, T2D is often difficult to reverse, so the transcriptome difference is higher than that in other states. There were 17 DEGs that met the DEGs screening criteria in all disease stages and had the same disease expression trend (Supplementary Table 1). Thus, these genes may play a central role in T2D progression. Moreover, 61% of the DEGs in the ND-IGT process were also identified in ND-T2D, indicating that the DEGs of the predisease progression, such as the inflammation-related genes IL1RL1 and IL6, may continue to affect disease progression, providing evidence that inflammation is a risk factor for disease progression. Similarly, in the process of IGT-T2D, in addition to inflammation-related genes, glucose transport-related genes such as SLC2A2 and SLC26A4 also had different expression levels, which show that the development of T2D into an irreversible state is related to the imbalance of the glucose transport system.

## 4. GO/KEGG Enrichment Analysis for ND-IGT-T2D DEGs and WGCNA

### 4.1. GO Enrichment Analysis

The degrees of ND-T2D progression were grouped according to upregulated and downregulated expression, after which GO enrichment analysis was performed (*p* < 0.01). For GO enrichment results, a total of 409 GO entries of upregulated genes were obtained, including 354 BP, 33 MF, and 22 CC, and 115 GO entries of downregulated genes were obtained, including 72 BP, 12 MF, and 31 CC.

**Figure 4:**
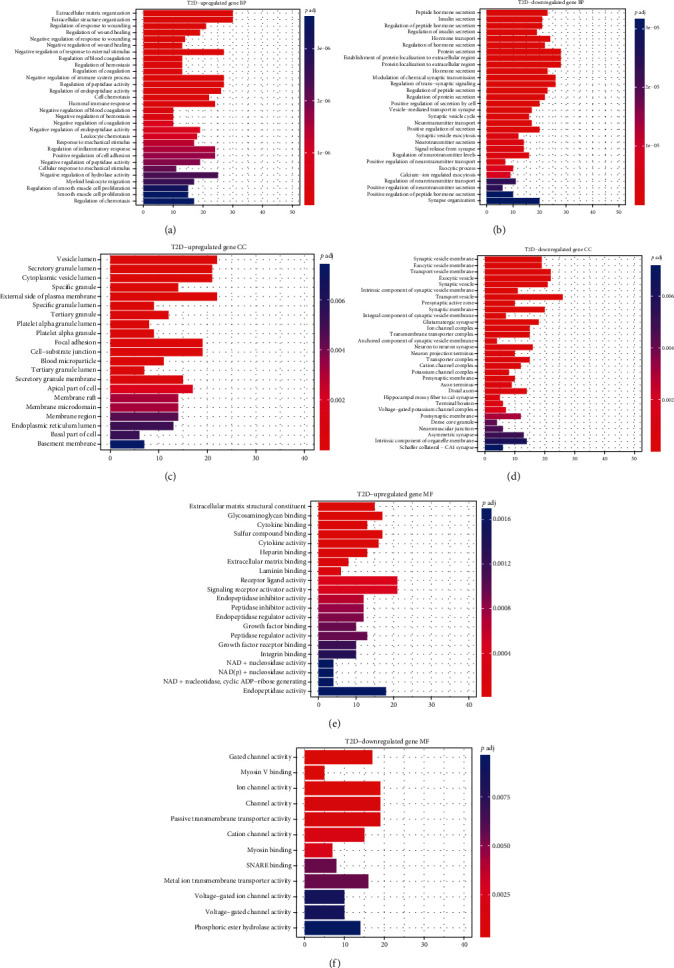
GO analysis of the up/downregulated DEGs involved in ND-T2D regarding biological process, cellular component, and molecular function.

The BP entry (*p*.adj < 0.01) of upregulated genes in ND-T2D mainly included regulatory response to injury, regulation of inflammatory response, regulation of cell adhesion, and acute inflammatory response regulation (Figure 4(a)), which may prove that the inflammatory response or inflammatory environment in the pancreatic islets is one of the reasons for the accelerated consumption of pancreatic islet *β*-cells. The BP entries enriched by downregulated genes (*p*.adj < 0.01) were mainly related to peptide hormone secretion, insulin secretion, protein secretion, etc. (Figure 4(b)). This result is consistent with the pathological phenotype of T2D; insulin secretion is reduced, and the ability of tissues to use insulin is downregulated.

GO-MF enrichment results showed that upregulated genes (*p*.adj<0.01) were mainly mapped to glycosaminoglycan binding, cytokine binding, cytokine activity, integrin binding, and other pathways related to immune stress, which matched the BP results (Figure 4(c)). Downregulated genes mainly enriched gated channel activity, ion channel activity, passive transmembrane transport protein activity, etc. (Figure 4(d)), all of which is related to the utilization of glucose by tissues due to downregulation, causing an imbalance in glucose transport. The enrichment analysis results for GO-CC (*p*.adj < 0.01) are shown in Figures 4(e) and 4(f).

In the ND-IGT process, the two physiological statuses are similar, and the number of DEGs is small. The GO enrichment results were included in ND-T2D and IGT-T2D, indicating that inflammation and decreased insulin secretion are two factors that accelerate the process in the early stage of T2D. For BP terms in the IGT-T2D process, upregulated genes were enriched in the humoral immune response, neutrophil activation-mediated immune response, immune response effector, lymphocyte-mediated immune response, and leukocyte-mediated immune response. This shows that during the progression of IGT to T2D, the inflammatory response that accelerates the consumption of pancreatic islet *β*-cells is an important factor leading to the progression of T2D. Downregulated genes are enriched in pathways such as insulin secretion, positive feedback of hormone secretion, positive regulation of insulin secretion, hormone transport, and lipid digestion and utilization. Contrary to the ND-T2D process, IGT-T2D downregulated genes are also enriched in lipid-related pathways, which shows that lipotoxicity may also affect the course of T2D. The results of the CC and MF items were similar to those of the ND-T2D enrichment analysis.

### 4.2. KEGG Enrichment Analysis

**Figure 5:**
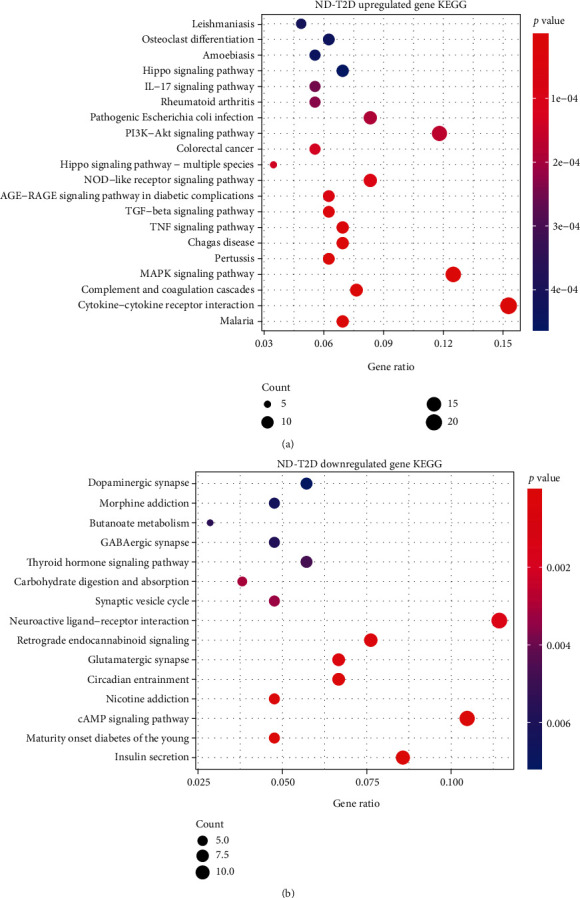
KEGG analysis of the up/downregulated DEGs involved in ND-T2D.

In the ND-T2D group, the upregulated expression gene enrichment (*p* < 0.05) was found in cytokine-cytokine receptor interaction, MAPK signaling pathway, Hippo signaling pathway, TGF-beta signaling pathway, TNF signaling pathway, diabetes AGE-RAGE signaling pathway, IL-17 signaling pathway, ECM-receptor interaction, NF-kappa B signaling pathway, Toll-like receptor signaling pathway, and NOD-like receptor signaling pathway (Figure 5(a)). The Hippo signaling pathway regulates cell proliferation and apoptosis to ensure that organ size is normal [32]. Studies have shown that the imbalance of this pathway may be one of the reasons why T2D patients are more likely to develop tumors [33], and the MAPK and TGF-beta signaling pathways have also been shown to be considerably related to cell proliferation. Cytokine-cytokine receptor interaction, Hippo signaling pathway, TNF signaling pathway, AGE-RAGE signaling pathway in diabetes, IL-17 signaling pathway, and ECM-receptor interaction, NF-kappa B signaling pathway, Toll-like receptor signaling pathways, and NOD-like receptor signaling pathways are all related to the onset of inflammation. Excessive levels of inflammatory factors in the cellular environment accelerate cell senescence and death, resulting in excessive consumption of abnormal pancreatic islet *β*-cells [34]. Downregulated genes were enriched in pathways such as insulin secretion, young diabetes, and cAMP signaling (Figure 5(b)). The results of KEGG and GO enrichment analyses are consistent, which enhances the credibility of the GO enrichment results and further shows that the cause of T2D is inflammation and insufficient insulin secretion. However, KEGG was enriched in the MAPK signaling pathway, TGF-beta signaling pathway, and other pathways related to cell proliferation, indicating that the cell cycle is also affected in the process of T2D. KEGG results of ND-IGT showed that the main signaling pathways affected were immune-related NF-kappa B signaling pathway, cytokine-cytokine receptor interaction, TNF signaling pathway, and lipolysis of fat cells. The regulatory pathways of serotonin were also enriched, which means that when the patients progressed to the IGT stage, the lipid metabolism disorder was a relatively apparent phenomenon. During the progression from the intermediate IGT state to the irreversible T2D state, the cAMP signaling pathway, insulin secretion, fat digestion and absorption, and other pathways indicated that the level of insulin secretion, cell growth, and the ability of the body to utilize lipids have declined.

### 4.3. WGCNA Analysis

The height cut-off value was set at 80, and 2 outlier samples were excluded in our analysis (Supplementary Figure 4).

**Figure 6:**
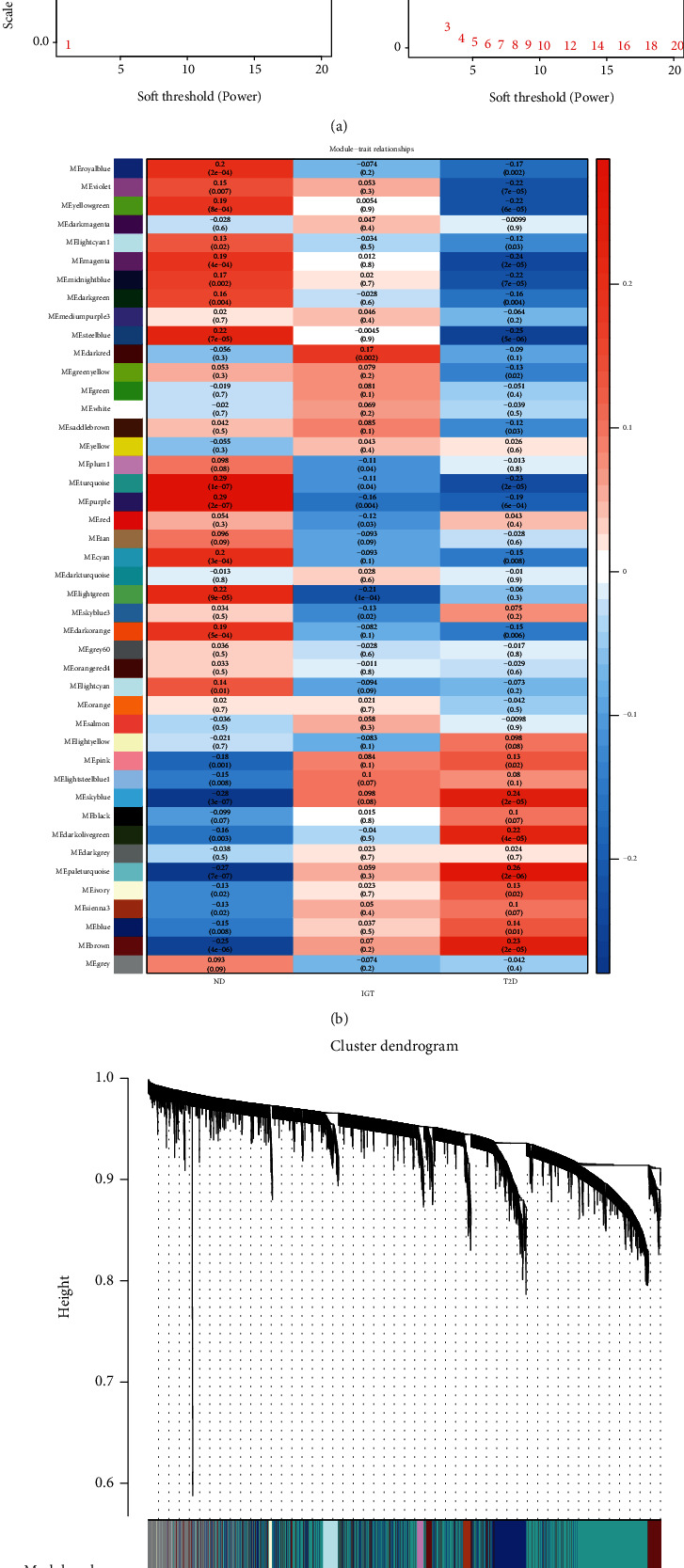
WGCNA in ND-IGT-T2D. (a) Analysis of network topology for various soft-thresholding powers. (b) Module–trait associations. Each row corresponds to a module, and each column corresponds to a trait. Each cell contains the corresponding correlation and *p* value. The table is color-coded by correlation according to the color legend. (c) Clustering dendrogram of genes, with dissimilarity based on topological overlap, together with assigned module colors.

Since scale independence reached 0.9 and average connectivity was high (Figure 6(a)), the soft threshold power *β* was set to 4 in subsequent analyses. Furthermore, we constructed a gene network and identified the modules. For cluster splitting, the soft thresholding power was set to 4, the minimum module size was set to 30, and DeepSplit was set to 2 (which implies medium sensitivity). Finally, 45 gene coexpression modules were constructed (Figure 6(b)).

We correlated the modules with clinical characteristics (ND/IGT/T2D) and searched for the most significant associations. The results of this analysis showed that the turquoise and purple modules were markedly correlated with T2D (Figure 6(c)).

We conducted GO and KEGG analyses of genes in the turquoise and purple modules (Supplementary Figures 5 and 6). The results of these analyses showed that regarding BP, the genes of the turquoise module were mainly enriched in synapse organization, and the genes of the purple module were mainly enriched in the regulation of protein secretion. As for CC, the genes of the turquoise module were mainly enriched in microtubules, and the genes of the purple module were mainly enriched in the extracellular exosome. Finally, regarding MF, the genes of the turquoise module were mainly enriched in tubulin binding, and the genes of the purple module were mainly enriched in calcium ion binding. We then performed KEGG analysis of the genes in the two modules and identified the module-regulated pathways. The results of this analysis showed that the purple module-regulated pathways included herpes simplex virus 1 infection and amino acid degradation, but the turquoise module was enriched for insulin resistance, glucagon signaling pathway, and insulin secretion.

The turquoise module contained 2597 genes. Using a gene significance (GS) over 0.2 and an MM over 0.8 as the cut-off criteria, 158 genes were identified as hub genes (Supplementary Table 2). Using GS > 0.2 and MM > 0.6 as the cut-off criteria, 156 genes were identified as hub genes from 225 genes in the purple module (Supplementary Table 2). There are genes that have been reported to be associated with T2D: TSPAN7, CNTN1, NOL4, NMNAT2, and TMEM196 in the turquoise module, and THNSL1, ZBED8, ZNF420, ZNF512, and KBTBD7 in the purple module.

The WGCNA results reinforce previous findings and suggest that inflammation and islet function are responsible for T2D because the hub genes are associated with the main T2D phenotype.

## 5. Active Compounds in GLQMW

This study is based on the fact that GLQMW can stabilize the blood glucose levels of patients with T2D in the long-term clinical practice of TCM, and even when the patient reduces the use of hypoglycemic drugs and exogenous insulin, it can ensure that the blood glucose level of the patients is maintained at a normal level. Therefore, in our study, we investigated the components of GLQMW, whether its compounds can have potential hypoglycemic effects. GLQMW is composed of five herbs: THF, FZ, FL, QM, and SY. The compounds in GLQMW that met the screening criteria were QM 1, THF 2, and FL 15 (two compounds, poricoic acid A, and poricoic acid B were selected to join the study based on literature reports [35]), FZ 16 (choose 5 have been reported, OB and DL meet the conditions but Caco-2 does not meet the screening criteria, add the drug screening results [36, 37]), and yams 16 (add a compound that does not meet the Caco-2 screening criteria [38]). A total of 50 compounds were included in our study (Supplementary Table 3).

## 6. Target of Compounds Prediction

**Figure 7:**
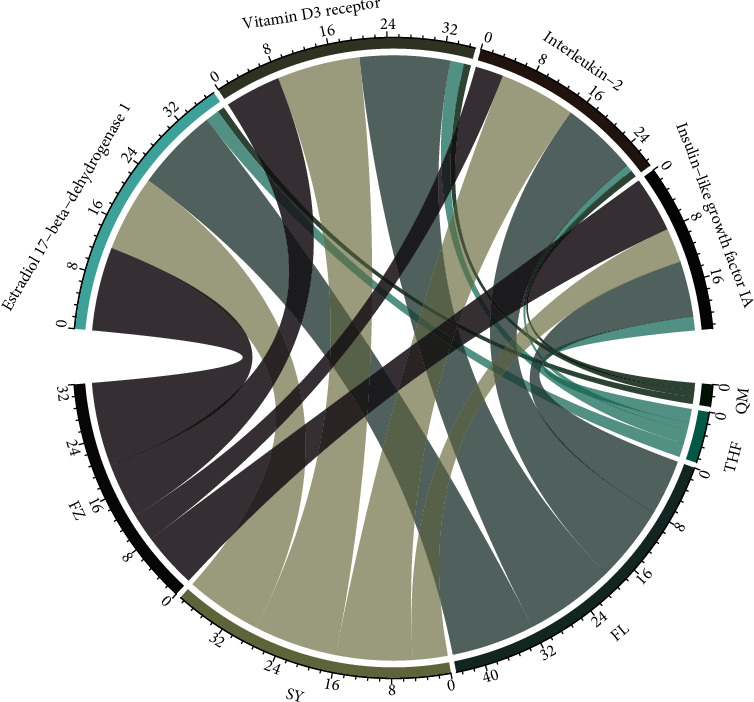
Correspondence between GLQMW and main target proteins.

The PharmMapper platform was used to input the 3D structure of the drug-like compound and predict the human target protein by matching the pharmacophore. In this study, the *Z*-score was used as the basis for judging the strength of the correlation between the compound and the target protein, and the predicted targets that met the standard (*Z* − score > 1.0) were included in further studies.  Figure 7 shows the correspondence between drug-like compounds and the predicted targets. We then performed a statistical analysis on these predicted targets. Several proteins, such as corticosteroid 11-b-dehydrogenase isoenzyme 1 (HSD11B1), estradiol 17-b-dehydrogenase 1, vitamin D3 receptor (VDR), insulin-like growth factor, glutathione S-transferase A1, SEC14-like protein 2, mineralocorticoid receptor, 72 kDa Type IV collagenase, oxysterol receptor LXR-alpha, and bile acid receptor, that are frequently targeted by the compounds may be the specific targets for GLQMW in patients with T2D.

## 7. GO/KEGG Enrichment Analysis for GLQMW Targets

**Figure 8:**
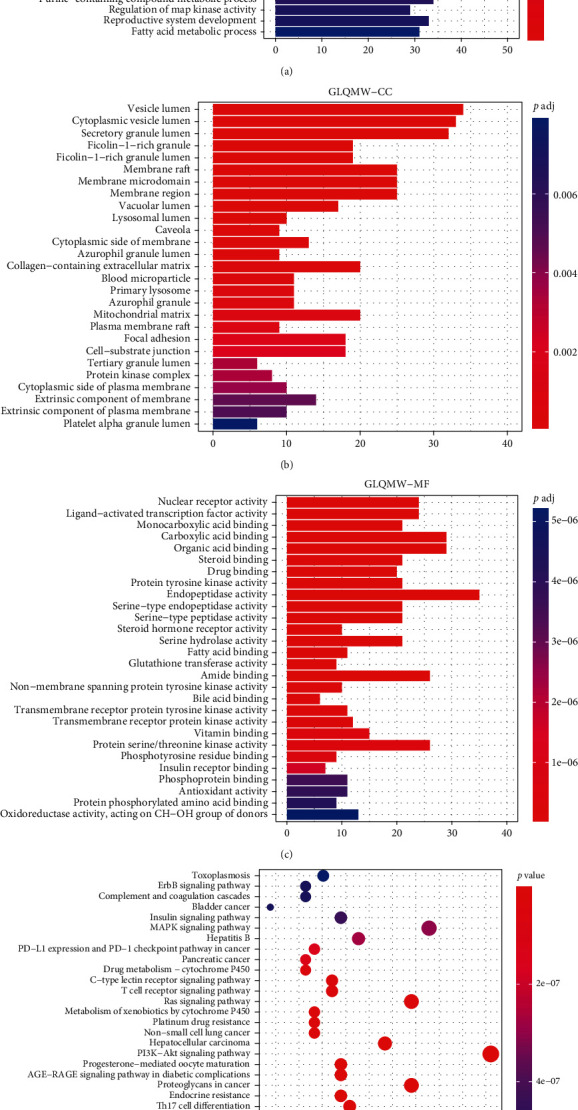
GO and KEGG analysis of the GLQMW target proteins.

GO enrichment analysis was performed on the target genes of GLQMW (*p*.adj < 0.05). The top 30 significantly enriched GO-BP terms are listed in Figure 8(a). The results clearly demonstrate that numerous targets are involved in various biological processes associated with fatty acid metabolism, regulation of MAP kinase activity, hormone-mediated signaling pathways, positive regulation of protein kinase B signal transduction, and response to oxidative stress. The GO-CC enrichment analysis results shown in Figure 8(b) demonstrated that GLQMW acts mainly on the cell membrane, plasma membrane, extracellular space, and ficolin-1 rich granular cavity, which are closely related to insulin secretion. The results of GO-MF enrichment analysis shown in Figure 8(c) demonstrate that GLQMW targets are involved in antioxidant activity, phosphotyrosine residue binding, protein serine/threonine kinase activity, bile acid binding, and insulin receptor binding.

KEGG analysis of the GLQMW target (*p* < 0.05), Figure 8(d), showed significant enrichment in inflammation-related pathways such as focal adhesion, chemokine signaling pathway, NF-*κ*B signaling pathway, NOD-like receptor signaling pathways, natural killer cell-mediated cytotoxicity, IL-17 signaling pathway, relaxin signaling pathway, and T-cell receptor signaling pathway. These pathways, as per our clinical observations, regulate the levels of related inflammatory factors in pancreatic islet tissues. Pathways related to glucose and lipid metabolism, such as those of type II diabetes, diabetic cardiomyopathy, endocrine and insulin resistance, insulin signaling, atherosclerosis, and adipocytokine signaling, play an important role in insulin resistance.

## 8. Network Analysis of GLQMW Active Compounds

**Figure 9:**
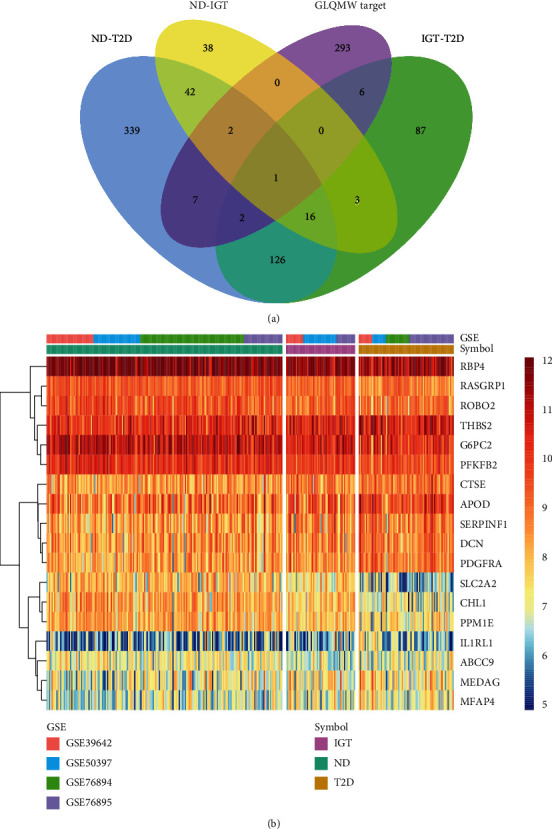
Venn diagram and heatmap of intersecting DEGs between process of ND-IGT-T2D and GLQMW target proteins. (a) Venn plots showing the intersecting targets between common DEGs in ND-IGT-T2D and GLQMW target proteins. (b) The expression level of intersecting DEGs in ND, IGT, and T2D.

The predicted target of GLQMW and the degrees of all stages of T2D were intersected, and 18 target proteins were identified (Figures 9(a) and 9(b)). The targets of GLQMW in ND-IGT are C1S, RBP4, and C1R. Targeted genes in IGT-T2D include KDR, HADH, HSD11B1, ALB, RBP4, KIT, HMOX1, PLAT, and CFB. In the T2D stage, the targeted genes were MET, HADH, FABP4, EGFR, C1S, RBP4, PLAU, C1R, EPHA2, EPHX2, TGFB2, and PLAT.

Hepatocyte growth factor (HGF) binds to MET, activating signal transduction and the RAS and PI3K/Akt signaling pathways [39] to regulate cell growth and the cell cycle. Fatty acid-binding protein 4 (FABP4), abundantly expressed in adipocytes, plays an important role in adipocyte differentiation and lipid metabolism [40]. FABP4 in the serum is responsible for the transport of free fatty acids and can affect the regulation of systemic insulin sensitivity [41, 42]. Retinol-binding protein 4 (RBP4) is present in the serum and can assist the liver in releasing retinol into the systemic circulation to meet tissue needs, but high concentrations of RBP4 in the serum are associated with an increased risk of T2D [43, 44]. Doctors use vitamin A analogs such as fenretinide to increase urine excretion, reduce the high expression of RBP4 caused by a high-fat diet, and restore insulin sensitivity [45]. Therefore, these may be the potential targets by which GLQMW can treat T2D.

**Figure 10:**
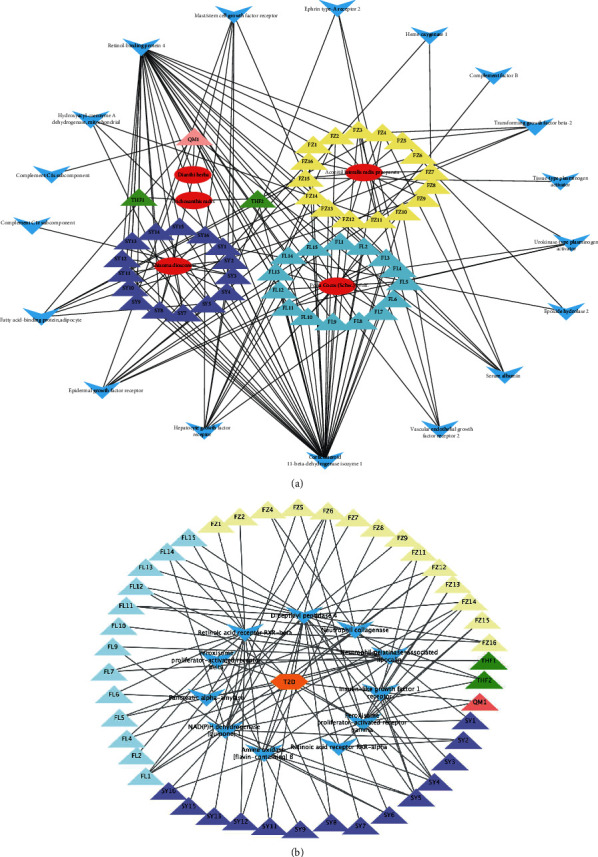
Interactions between herbs and target proteins for compounds. (a) The ovals represent herbs; the triangles represent the compounds of herbs, and different colors represent the source of the compound; the blue darts represent the intersecting target proteins between GLQMW and process of ND-IGT-T2D. (b) Hexagons represent T2D; the triangles represent the compounds of herbs, and different colors represent the source of the compound; blue darts represent clinical drug targets.

In our research, small-molecule compounds (Figure 10(a)) such as FZ16 and SY3, having multiple targets and a potential therapeutic effect on them, were included in the follow-up analysis to further interpret their mechanisms of action. Based on previous results, we analyzed the compounds with the target proteins of clinical drugs (Figure 10(b)) and found that THF2, FZ10, QM1, FZ16, and SY3 also act on the targets of drug action.

## 9. Similarity Analysis of Active Compounds

**Figure 11:**
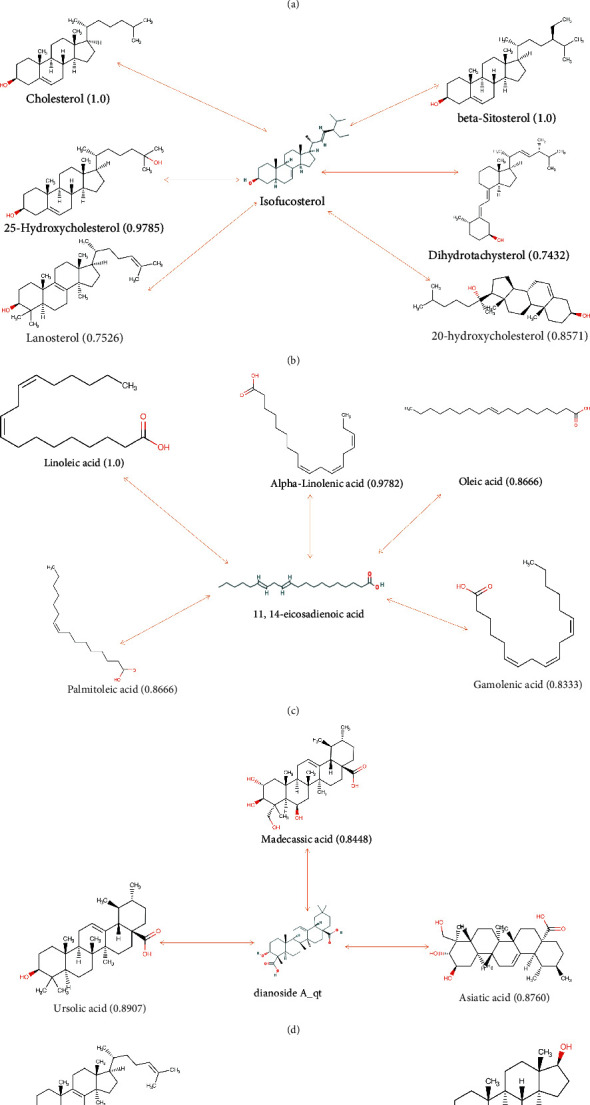
Drug similarity between compounds and drugs of DrugBank. The two-way line represents the comparative relationship between the compound and the drug, and the numbers represent the drug similarity. (a) drug similarity of (-)-taxifolin; (b) drug similarity of isofucosterol; (c) drug similarity of 11,14-eicosadienoic acid; (d) drug similarity of dianoside A_qt; (e) drug similarity of spinasterol.

Based on the RDKit tool, the small-molecule compounds obtained were compared with all small-molecule drugs released from the DrugBank database, and the top 10 drug compounds with similarity were output. The drug similarities of (-)-taxifolin, dihydromyricetin, silibinin, hesperetin, epicatechin, and epigallocatechin gallate were 0.9958, 0.8417, 0.8119, 0.7952, and 0.7227, respectively (Figure 11(a)). Dihydromyricetin, a research drug, can inhibit oxidative stress, enhance neuroprotection, and reverse cognitive impairment caused by T2D and abnormal glucose and lipid metabolism in mouse models [46]. Silibinin has PPAR*γ* agonist properties, and PPAR*γ* is the specific target of thiazolidinedione in the treatment of T2D [47]. Hesperetin can regulate glucose metabolism by changing the activity of glucose-regulating enzymes and reducing lipid levels in the serum and liver [48]. Epicatechin works by increasing the expression of antioxidant enzymes, reversing the production of reactive oxygen species (ROS) in skeletal muscle and regulating autophagy involving mitochondria. Epicatechin can also increase the oxidation of muscle lipids and stimulate insulin-resistant skeletal muscle absorption of glucose [49]. Epigallocatechin gallate has a significant insulin-like effect on erythrocyte membrane-bound AChE to achieve a therapeutic effect [50]. The similarities between isofucosterol and cholesterol, beta-sitosterol, 25-hydroxycholesterol, 20-hydroxycholesterol, lanosterol, and dihydrotachysterol were 1.0, 1.0, 0.9785, 0.8571, 0.7526, and 0.7432, respectively (Figure 11(b)). Beta-sitosterol has been shown to lower blood sugar in clinical trials and T2D model mice [51], inhibit the serine phosphorylation of IRS induced by hyperlipidemia, and restore the expression of GLUT4 in adipose tissue [52]. Kim et al. showed that 20-hydroxycholesterol can inhibit fat formation in mice through a hedgehog-dependent mechanism [53], which is related to alleviating insulin resistance. 11,14-eicosadienoic acid is highly similar to linoleic, alpha-linoleic, oleic, palmitoleic, and gamolenic acids (Figure 11(c)). Linoleic acid and alpha-linoleic acid, essential fatty acids, have been shown to improve liver fat deposition and insulin resistance [54] and inhibit the expression of IL-1*β* and Toll-like receptors, exerting anti-inflammatory effects [55]. Gamolenic acid inhibits the expression of intercellular adhesion molecule-1 (ICAM-1) and monocyte chemoattractant protein-1 (MCP-1) to reduce the degree of inflammatory response, and it also affects the aggregation of the extracellular matrix (ECM) in patients with diabetic nephropathy [56]. Drugs with high similarity to dianoside A_qt were madecassic acid, ursolic acid, and asiatic acid (AA) (Figure 11(d)). The anti-inflammatory effect of madecassic acid regulates the dynamic balance of Th17/Treg cells because the low proportion of Treg cells leads to chronic inflammation [57]. Madecassic acid restores the Th17/Treg balance by regulating the PPAR*γ*/AMPK/ACC1 pathway, and therefore can reduce local inflammation [58]. Ursolic acid inhibits *α*-amylase and *α*-glucosidase activity by binding to their inactivation sites and has been shown to rapidly reduce the blood glucose concentrations 2 h after a meal in animal models [59]. AA reduces the decomposition of glycogen to glucose and is released into the blood by inhibiting the translation of GSK-3*β* and glucose 6-phosphatase [60]. Particularly, AA prevents islet dysfunction by downregulating islet fibrosis caused by fibronectin [61]. In the results of spinasterol (Figure 11(e)), dihydrotachysterol, a vitamin D analog has proven that vitamin D and its analogs can be used to assist in the treatment of T2D [62]. Androstenediol can increase PPAR-*γ* DNA-binding activity, activate the PPAR-*γ* pathway to inhibit IL-6 and iNOS gene expression, and achieve the purpose of slowing down local inflammation [63]. 20-hydroxycholesterol has a regulatory function of circulating lipid levels. These compounds may serve as the basis for GLQMW in T2D treatment.

## 10. Molecular Docking and LIGPLOT

**Figure 12:**
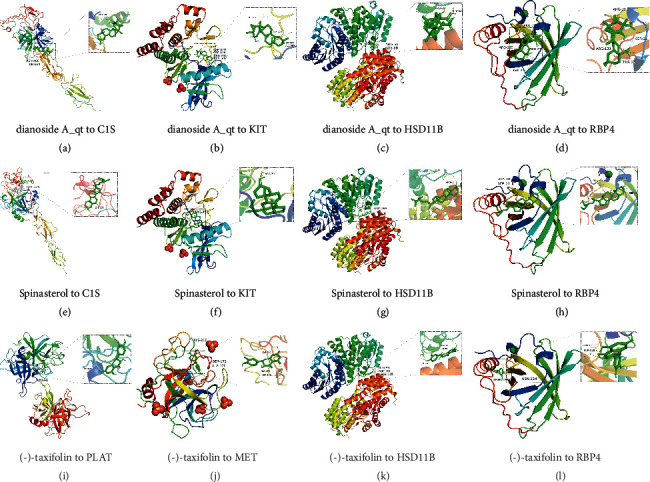
The docking mode and interactions between compounds and target proteins.

The docking results of the active compounds with C1S, HSD11B, KIT, MET, PLAT, and RBP4 are presented in Supplementary Table 4. Compound (-)-taxifolin showed the lowest binding energy with HSD11B (−7.5 kcal/mol), MET (−7.1 kcal/mol), PLAT (−7.6 kcal/mol), and RBP4 (−6.2 kcal/mol). Dianoside A_qt was found to have the lowest binding energy with C1S (−8.4 kcal/mol), HSD11B (-8.8 kcal/mol), KIT (−8.6 kcal/mol), and RBP4 (−7.5 kcal/mol). Spinasterol had the lowest binding energy with C1S (−9.2 kcal/mol), HSD11B (−8.5 kcal/mol), and KIT (−9.2 kcal/mol). Binding energy analyses showed that the active compounds in GLQMW formed stable conformations with the target proteins. The docking analysis between the selected compounds and target proteins is shown in Figure 12. Compound dianoside A_qt bound to C1S, forming hydrogen bond interactions with residues Asn466 and Thr684; HSD11B, forming hydrogen bond interactions with residues Arg66 and Lys68; KIT, forming hydrogen bond interactions with residues Asp820, Asp816, Arg815, and Ala597; and RBP4, forming hydrogen bond interactions with residues Arg139, Ser21, Arg19, and Thr23. When binding to HSD11B, (-)-taxifolin formed hydrogen bonds with residues Glu94, Arg66, and Gln105. Furthermore, when binding to RBP4, (-)-taxifolin formed hydrogen bond interactions with residues Asn124, Thr128, MET, Lys210, Ser172, and Ala170 (Figure 12). In addition, when binding to RBP4, C1S, HSD11B, and KIT, spinasterol formed hydrogen bond interactions with residues Ala18, Ser21, Lys575, Arg66, and Arg791, respectively. The ligand plot results, Supplementary Figure 7, show the hydrophobic interaction sites of the target protein.

## 11. Discussion

With societal development and improvements in living standards, T2D has become a major chronic disease that threatens human health because of its gradually increasing incidence. Currently, physicians tend to use drugs for T2D control, supplemented by diet and rhythmic exercise. However, due to the continuous increase in insulin resistance in the body, weakening of drug sensitization, and increase in drug resistance in the body, it is often difficult for T2D patients in the later stage to maintain normal blood glucose ranges. The patient's quality of life significantly deteriorates due to constant medication and insulin injections, resulting in decreased treatment efficacy. Therefore, it is necessary to develop a new clinically effective treatment strategy. TCM is a medical discipline that has been summarized and developed by the Chinese nation in long-term practice. GLQMW, which are briefly recorded in the “JinGuiYaoLue,” have the function of treating T2D, but no scholars have summarized and discussed the effectiveness and treatment mechanism of TCM prescription. It is generally believed that the developmental trend of type 2 diabetes is ND-IGT-T2D, in which IGT is an intermediate state and not a stage of irreversible diabetes damage [3]. Therefore, if the T2D stage can be reverted to the IGT stage through adjustment of self-living habits, the T2D patient can then more appropriately achieve the purpose of curing T2D. In our study, during IGT, we found that inflammation and lipid metabolism disorders played a significant role in the process of ND-IGT. For the treatment of patients with IGT, focus on fighting inflammation and adjusting our diet to avoid high fat and sugar intake is mandatory. The progression of IGT to T2D involves abnormalities in *β* cells, which mainly include a significant decrease in the number of pancreatic *β*-cells and a decrease in the function of *β*-cells to secrete and synthesize insulin. The decrease in the number of pancreatic *β*-cells is related to the disorder of the *β*-cell cycle [64], but the decrease or loss of islet *β*-cell function is associated with islet *β*-cell dedifferentiation, glucotoxicity caused by high concentrations of glucose [65], lipotoxicity caused by free fatty acids and their related products [6], and chronic inflammation in islet tissue [66] to a certain extent. Accordingly, doctors can design rational drug regimens according to the etiology of patients with T2D.

Based on the results of gene expression profiling and WGCNA, we derived the disease patterns and key targets of T2D. IL1RL1, a membrane receptor whose expression is continuously upregulated during the progression of T2D, can bind to IL-33 to activate the TH2 inflammatory response and eosinophilia [67]. IL-33 is also markedly upregulated in the T2D stage, which can competitively inhibit angiotensin II and phenylephrine, and over-activate the NF-*κ*B and MAPK signaling pathways [68]; thus, the IL-33 and IL1RL1 complex can sustainably induce local inflammation in islet tissue during ND-T2D progression. Similarly, IL-17, produced by Th17 cells, can effectively mediate neutrophil mobilization and acts as a proinflammatory factor that causes an inflammatory storm and enhances the intensity of the inflammatory response [69]. However, high expression levels of IL-17 cause chronic inflammation in pancreatic islets [70]. According to the results of WGCNA, RRAGD, PPM1E, PFKFB2, and CHL1 were hub genes in the major modules of T2D, and the expression of these genes continued to be downregulated with the prolongation of the disease course. RRAGD, a monomeric guanine nucleotide-binding protein, plays a crucial role in the regulation of the mTORC1 signaling cascade, promoting growth in response to growth factors, energy levels, and amino acids [71]. When the expression of RRAGD is reduced, mTORC1 signaling pathway function is dysregulated, which affects insulin sensitivity [72]. PPM1E is in cAMP-activated protein kinase (AMPK) phosphatases and is a potential antidiabetic drug target [73]. PFKFB2 is one of the key enzymes of glycolysis, and its low expression will inevitably reduce glucose utilization, while CHL1 affects the migration and cell cycle of islet *β*-cells. Therefore, the results in this study further explain the pathogenesis of T2D from the molecular mechanism and provide new strategies for treatment, such as specifically reducing the degree of inflammation in the pancreatic islets. According to the results of the Connectivity map (CMap) database (Supplementary Figure 8), the HDAC inhibitor [74] may be a promising drug that can antagonize the ND-T2D process (Supplementary Table 5), and the spleen tyrosine kinase (SYK) inhibitor, fostamatinib [75], can reverse the progression of IGT-T2D (Supplementary Table 6).

In the results of network pharmacology, the main targets of the active ingredients of GLQMW were HSD11B1, VDR, TGR5, FXR, and RBP4, all of which mainly related to T2D. Such finding may be the basis for the therapeutic effectiveness of GLQMW. According to the GO/KEGG results of GLQMW targets, in addition to inflammation-related and diabetes phenotype-related pathways, GLQMW was shown to be involved in pathways related to cell proliferation, such as the MAPK, JAK-STAT, Ras, and PI3K-Akt signaling pathways. GLQMW may restore the number of *β*-cells and restore islet function based on its effect on cell proliferation. This is in line with the clinical observation that GLQMW is effective in T2D patients with failed insulin control; based on anti-inflammatory and insulin resistance properties, GLQMW stimulates the proliferation of *β*-cells to achieve therapeutic purposes. In the results of drug similarity analysis, we found that compounds in GLQMW, including spinasterol, isofucosterol, dianoside A_qt, 11,14-eicosadienoic acid, and (-)-taxifolin, have potential therapeutic functions in T2D. We can isolate compounds for further experimental validation to determine whether they can be used as parents for new T2D drugs. Therefore, in the future, purified natural compounds can be prepared with a composition ratio similar to that of TCM prescriptions to replace existing decoction application methods [76]. Although some of the compounds involved in this study have demonstrated pharmacological activity, the efficacy of GLQMW in T2D has not been fully explained. In order to further improve the reliability of the study, we still need to comprehensively verify the effectiveness of GLQMW from cells, animals, and clinics, and enhance its efficacy.

## 12. Conclusion

GLQMW treat T2D by anti-inflammatory and restoring islet cell function, and its potential therapeutically active compounds are (-)-taxifolin, dianoside A_qt, isofucosterol, 11,14-eicosadienoic acid, and spinasterol.

## Data Availability

The data in this study were obtained from public databases.

## Supplementary Materials

Supplementary Materials Supplementary figures and tables show transcriptome profiles of different stages of diabetes, protein molecular forces, and CMap predictions of diabetes-reversing drugs.
