# Supplementary material for: Network Pharmacology and Transcriptomics Reveal the Mechanism of GuaLouQuMaiWan in Treatment of Type 2 Diabetes and Its Active Small Molecular Compound
Source: J Diabetes Res. 2022 Oct 6;2022:2736504. doi: 10.1155/2022/2736504 (PMC9560855; doi:10.1155/2022/2736504)
Supplement: Supplementary Materials — Supplementary figures and tables show transcriptome profiles of different stages of diabetes, protein molecular forces, and CMap predictions of diabetes-reversing drugs. [file 2736504.f1.zip › supplementary figure (1).docx]

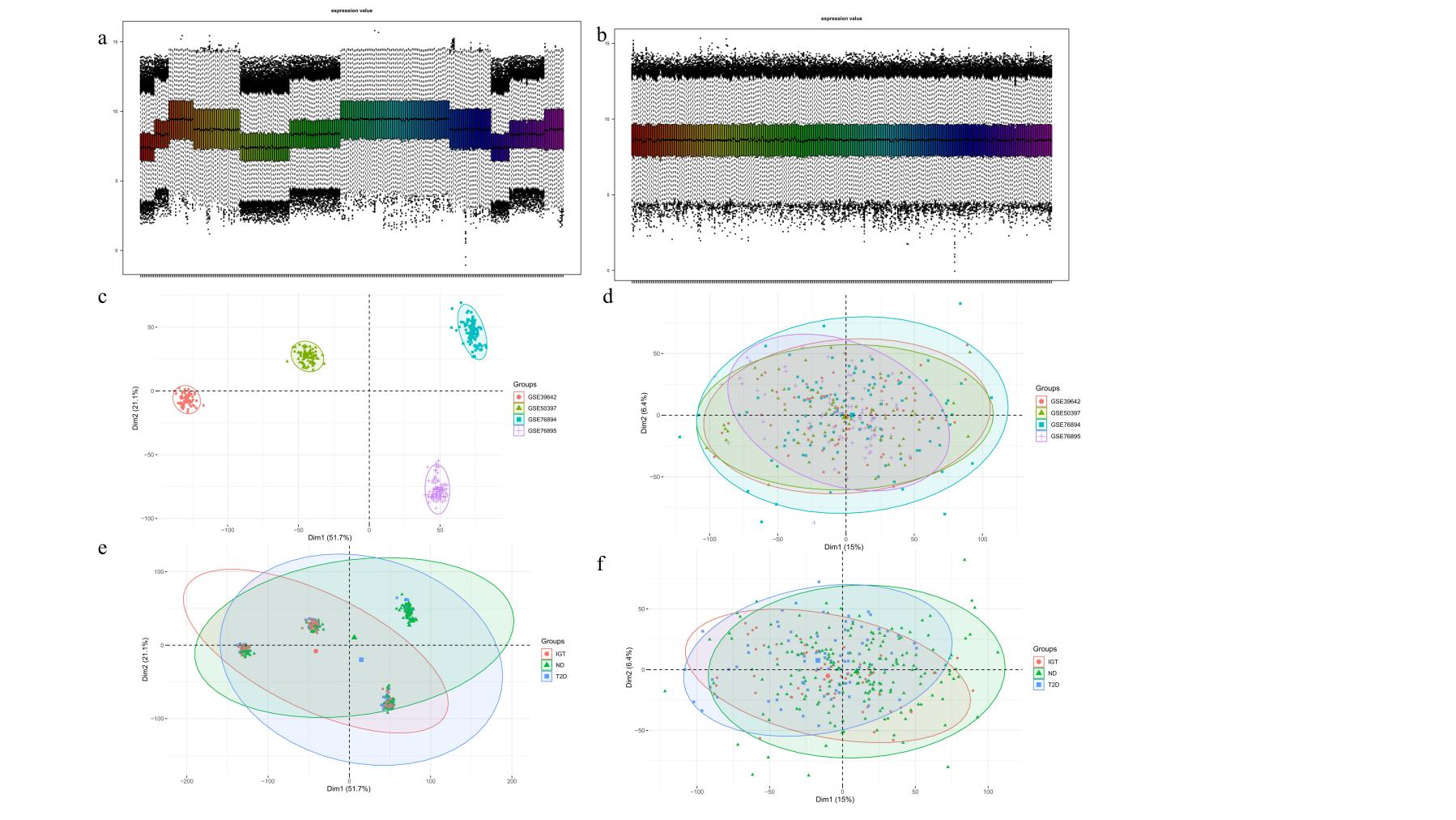


Supplementary Figure 1| Removal of batch effects in different datasets and PCA analysis


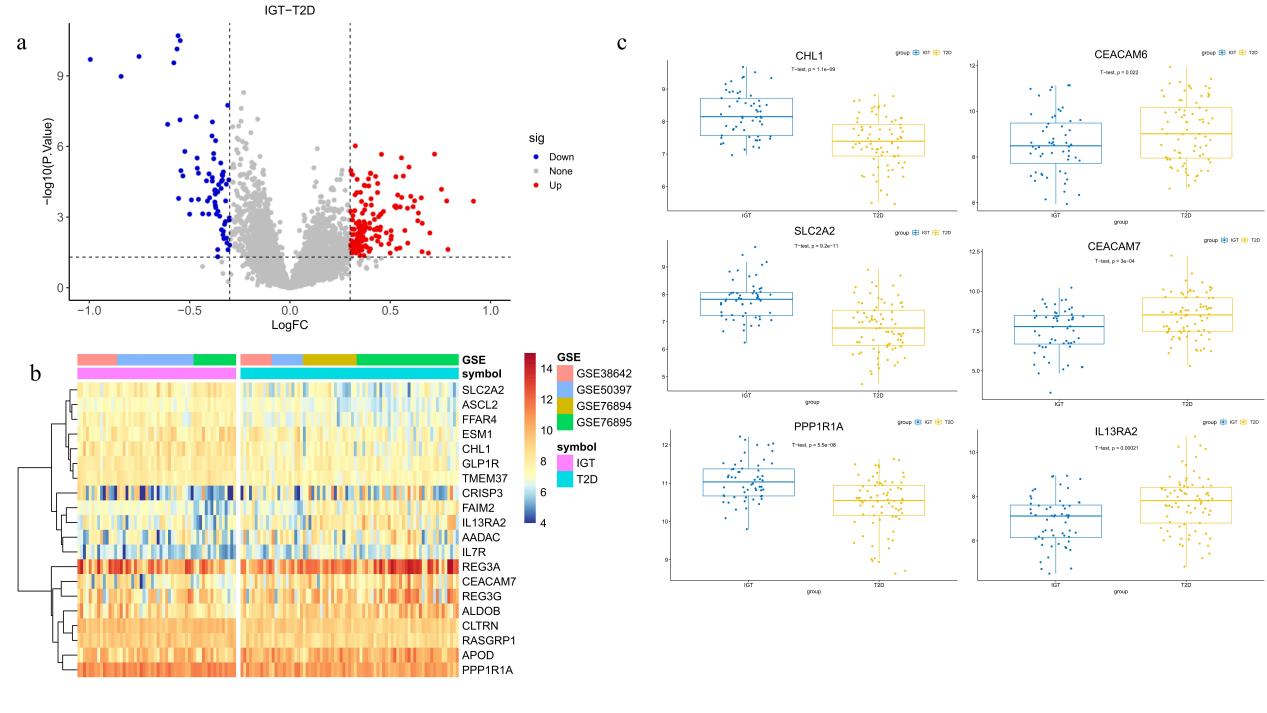


Supplementary Figure 2 | Volcano plots, heatmaps, and gene expression profiles of Degs in IGT-T2D.


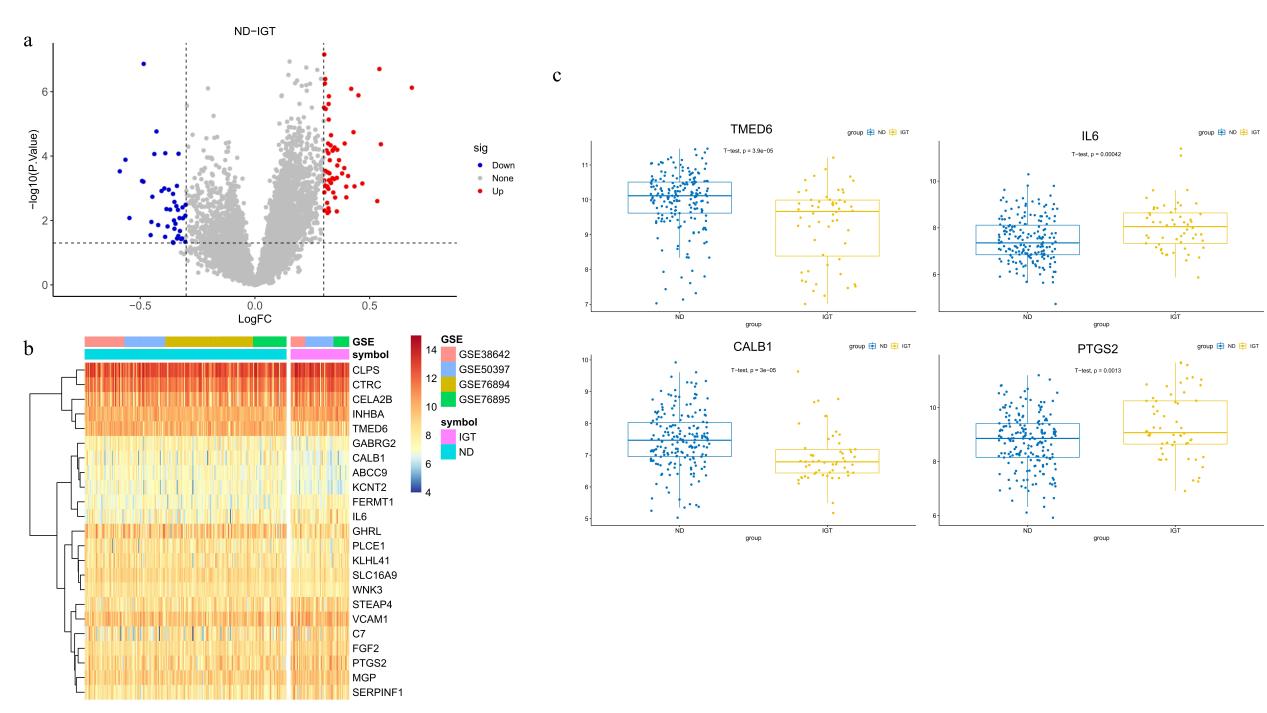


Supplementary Figure 3 | Volcano plots, heatmaps, and gene expression profiles of Degs in ND-IGT.


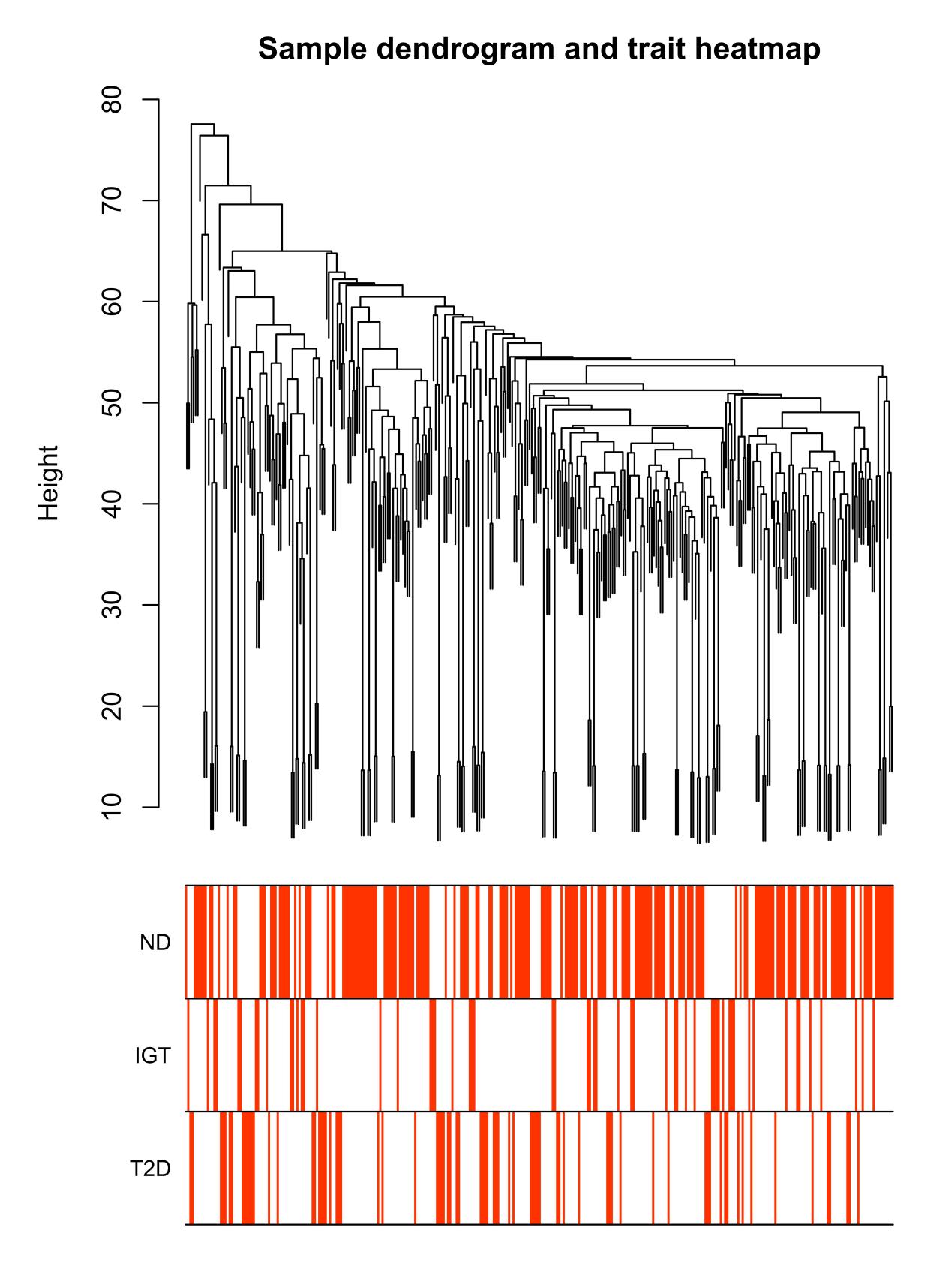


Supplementary Figure 4 | Clustering dendrogram of samples based on their Euclidean distance.


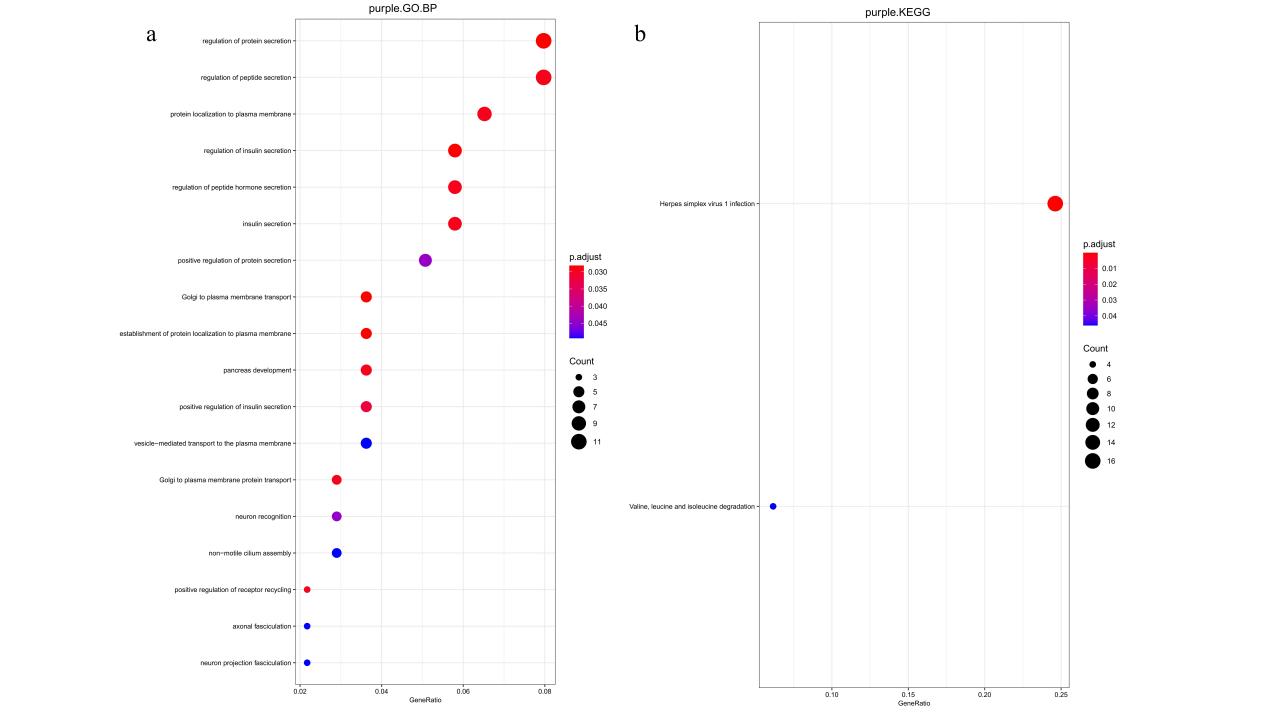


Supplementary Figure 5 | GO and KEGG analysis of the genes involved in the purple module


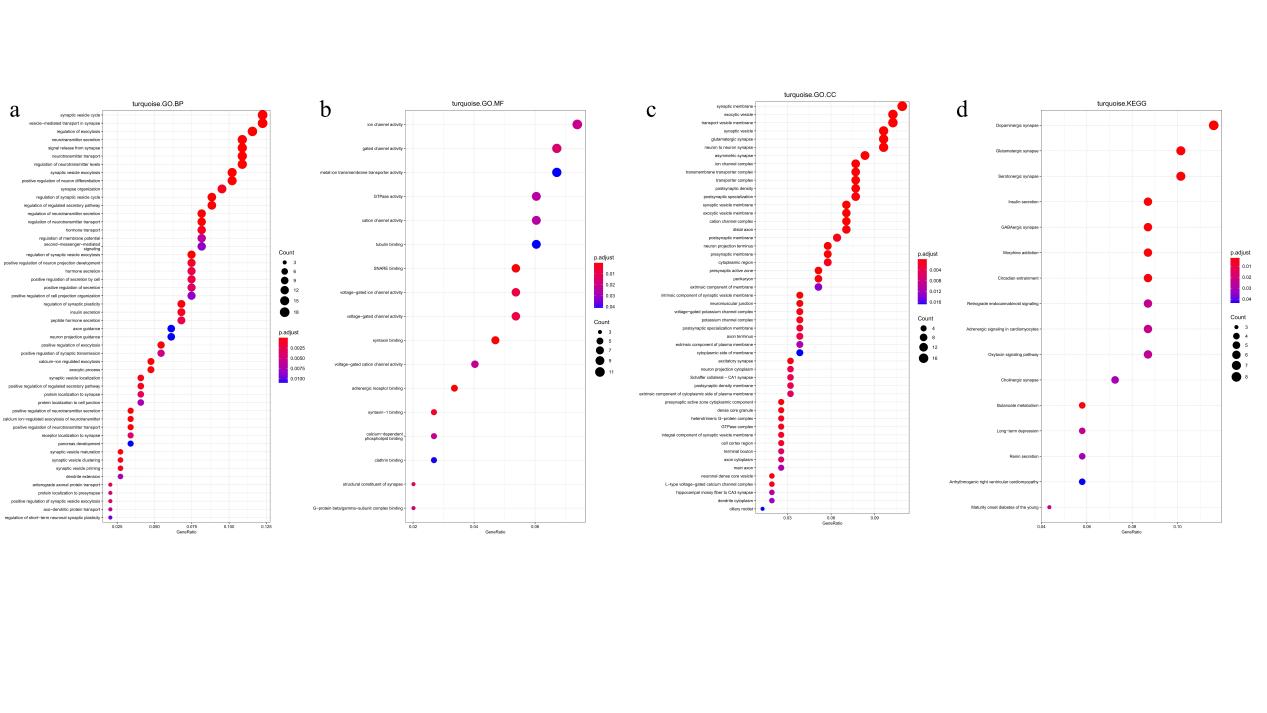


Supplementary Figure 6 | GO and KEGG analysis of the genes involved in the turquoise module


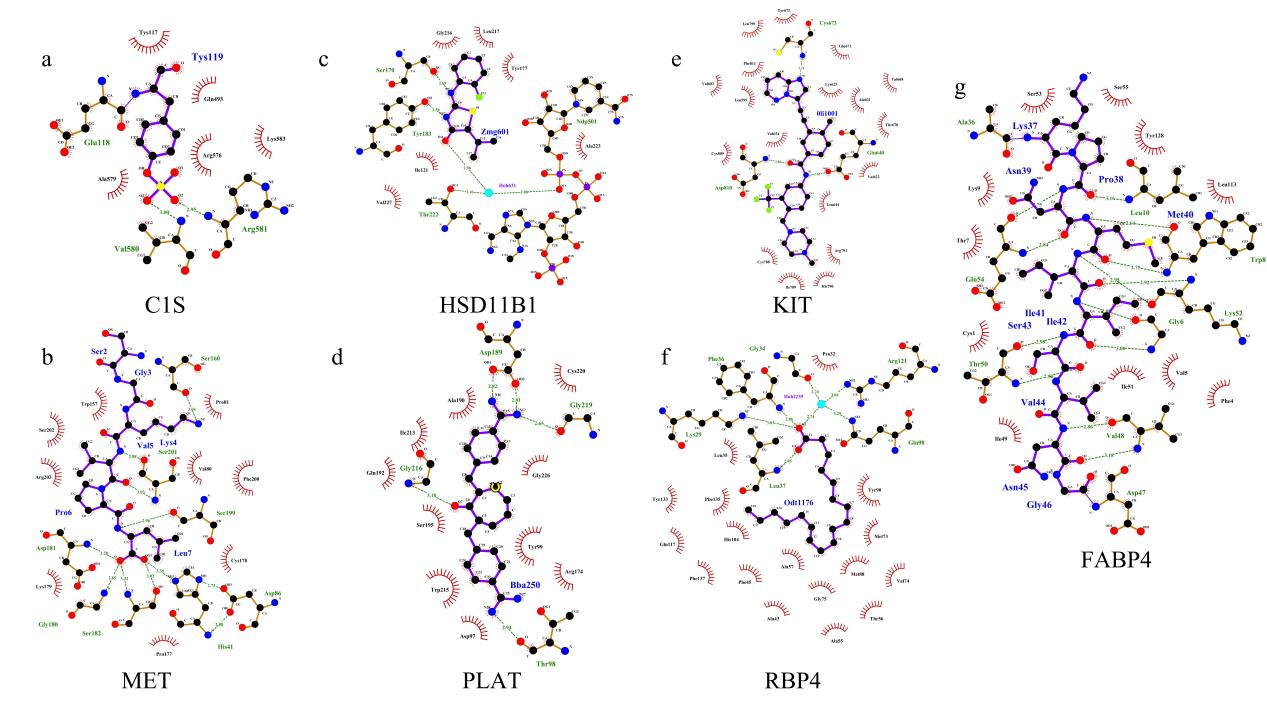


Supplementary Figure 7 |The ligplot mode of target proteins.


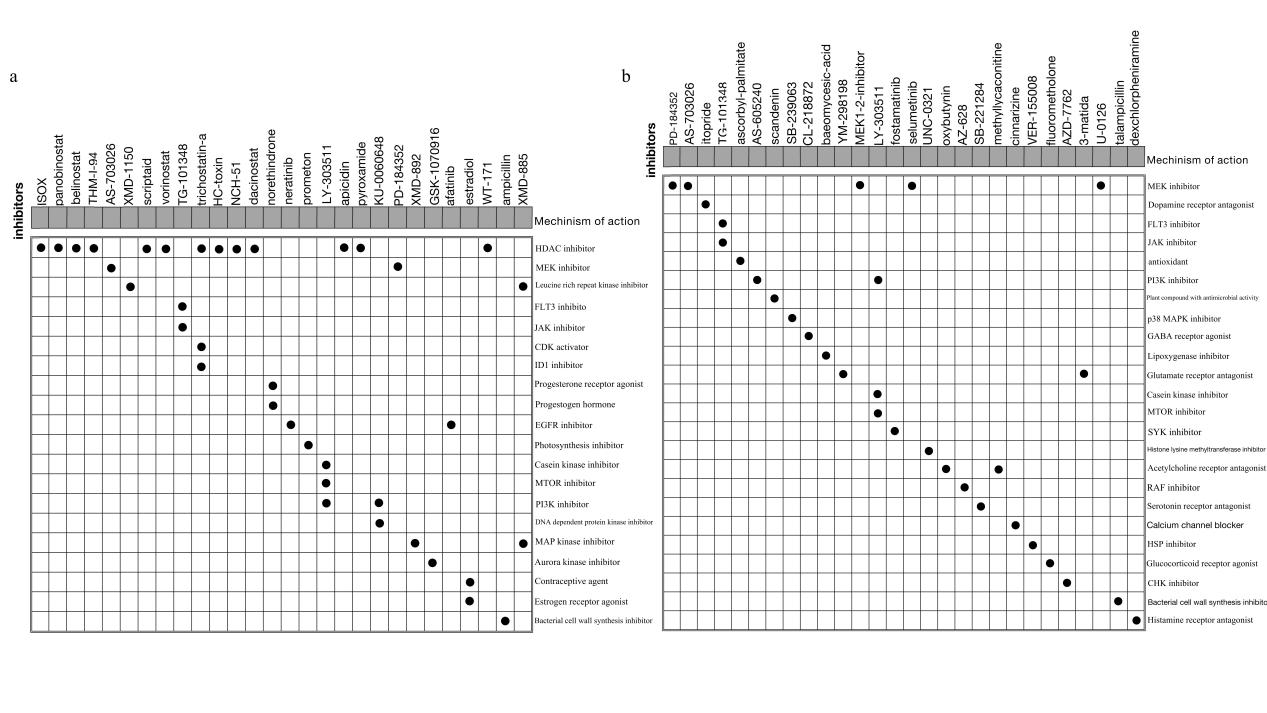


Supplementary Figure 8| CMap database analysis identifies potential cancidate small molecular drugs targeting the Degs between high- and low-groups.
