# Supplementary material for: Network Pharmacology and Transcriptomics Reveal the Mechanism of GuaLouQuMaiWan in Treatment of Type 2 Diabetes and Its Active Small Molecular Compound
Source: J Diabetes Res. 2022 Oct 6;2022:2736504. doi: 10.1155/2022/2736504 (PMC9560855; doi:10.1155/2022/2736504)
Supplement: Supplementary Materials — Supplementary figures and tables show transcriptome profiles of different stages of diabetes, protein molecular forces, and CMap predictions of diabetes-reversing drugs. [file 2736504.f1.zip › supplementary Table.docx]

Supplementary Table 1 Trends of Degs in ND-IGT-T2D

| Degs expression profile | Gene | number |
| --- | --- | --- |
| In ND-IGT, IGT-T2D and ND-T2D, meet the Degs criteria and the consistent trend | APOD, PDGFRA, SERPINF1, CTSE, IL1RL1, DCN, MEDAG, THBS2, MFAP4, ROBO2, RASGRP1, RBP4, PFKFB2, G6PC2, PPM1E, CHL1, SLC2A2 | 17 |
| Consistent trend in ND-IGT and IGT-T2D | ANXA10, APOD, CHL1, CTSE, DCN, G6PC2, IL1RL1, MEDAG, MFAP4, PDGFRA, PFKFB2, PPM1E, RASGRP1, RBP4, ROBO2, SERPINF1, SLC2A2, THBS2 | 18 |
| In ND-IGT and ND-T2D, meet the Degs criteria and the consistent trend | ACVR1C, ADAMTS1, ANXA1, APOD, ARL14, C1R, C1S, C7, CHL1, CNTN4, CTSE, CXCL12, CXCL17, DCN, FERMT1, FGF2, FGF9, FRZB, G6PC2, GABRG2, GHRL , IL1RL1 , IL6 , INHBA , KLHL41 , LAMC2 , LINC00643, MEDAG, MFAP4, MGP, MYH10, NKX2-2, NR0B1, NSG1, OTULINL, PDGFRA, PFKFB2, PLCE1, PPM1E, PTGS2, RASGRP1, RBP4, RFX6, ROBO2, RTL5, SCAPER, SERPINF1, SLC16A9, SLC2A2, SLC4A8, STEAP4, STXBP5L, SVOP, SYT14, TAGLN3, TFPI2, TGM2, THBS2, TMED6, VCAM1, VWDE | 61 |
| In IGT-T2D and ND-T2D, meet the Degs criteria and the consistent trend | ZMAT4, WSCD2, VCAN, VAV3, VAT1L, UNC5D, TTC7B, TMEM37, THBS2, TGFBR3, TCN1, SYT7, STK17B, SRGN, SPON1, SMAD9, SLPI, SLIT2, SLFN11, SLC2A2, SLC26A4, SLC25A34, SLC24A2, SLC16A7, SLAMF7, SERPINF1, SERPINB9, SEMA3C, SCN7A, SAMSN1, S100A8, RPH3A, ROBO2, RND3, RGS1, REG3G, RBP4, RASGRP1, RARRES1, PTX3, PTPRC, PPP1R1A, PPM1E, PON1, PMAIP1, PLAT, PLA2G7, PLA1A, PIGR, PFKFB2, PDGFRA, PDGFC, PDE1A, PAQR5, PAPPA, NPC1L1, NNMT, NKX6-1, NEXMIF, NECTIN3, MTUS2, MPZL2, MMP1, MGST1, MFAP4, MEDAG, LIPG, LCP1, LAPTM5, LAMP3, KYNU, KCNK16, KCNJ16, KCNH8, KCNG3, ITGA2, IRF8, IL7R, IL33, IL1RN, IL1RL1, IL1R1, IL13RA2, HSD17B2, HS6ST2, HLF, HK2, HHATL, HCN4, HADH, GRIA4GPR183, GPM6A, GPD1, GLP1R, GBP3, G6PC2, FGL1, FGB, FFAR4, FCER1G, FAM107A, FAM102B, FAIM2, ERMN, ENTPD3, ELFN1, DLGAP1-AS1, DKK3, DIO2, DHRS2, DDX3Y, DCN, CYP1B1, CXCL8, CTSV, CTSE, CRYAB, CRP, CRISP3, CLTRN, CHST4, CHL1, CH25H, CEACAM7, CEACAM6, CD53, CASR, CAPN13, C3, C15orf59, ATP2A3, ASCL2, ARG2, AREG, APOD, APCDD1L, ALDOB, ADGRG6, ADCYAP1, ADAMTS17, ADAM28, ABI3BP, ABCC8, AADAC | 145 |
| In ND-IGT and IGT-T2D,  meet the Degs criteria but with opposite trends | ABCC9 | 1 |

| Module | Gene | MM | GS | Num |
| --- | --- | --- | --- | --- |
| purple | AASDH, ABAT, ABHD10, ACOT13, ACVR1C, AIG1, ALDH9A1, AMPH, ANO5, APH1B, APOO, ARL6, ATP6V1D, BBS2, BBS9, C11orf49, C12orf60, C17orf80, C3orf14, CD200, CETN3, CHM, CHST9, CLCN4, CLIP4, CNTN4, COL4A3BP, CYP2U1, DCBLD1, DMXL2, DNAJC24, DUSP19, DZIP3, ELAVL4, ELMO2, ELOVL4, ELP4, ENO2, ETFDH, EXOC2, FAM120C, FAM200B, FBXL2, FOCAD, FOSL2, GCNT1, GFM2, GKAP1, GNPDA1, GPR158, GRK3, HADH, HCFC2, HDHD2, HOOK1, HSDL1, HSPA4L, HTR1F, ICE2, IGIP, ISL1, KBTBD3, KBTBD6, KBTBD7, KCNB2, KIF1BP, KLHL42, KRT222, LYRM7, MBLAC2, MEGF9, MEIS2, MICU3, MLH1, MOB1B, MTERF2, MTMR7, MYH10, N6AMT1, NARS2, NCALD, NDN, NEBL, NEUROD1, NKIRAS1, NKX2-2, NOL4, NPTX, NSF, OXCT1, PDZD2, PEX1, PIGV, PLCB4, POT1, PPID, PPP3CB, PTCD2, RAB3C, RAB9B, RABGAP1, RCBTB2, RCOR3, RFX6, RHOT1, RNGTT, ROBO2, SCRN3, SENP8, SH3GL2, SLC16A9, SLC46A1, ST18, SUCLA2, SUOX, SYT14, TBC1D32, TCEANC, THNSL1, TMEM17, TMEM196, TMEM260, TMEM60, TMOD2, TOGARAM1, TRIM59, TSPAN2, VDAC3, VPS37A, WDR7, WNK3, ZBED8, ZFP2, ZKSCAN1, ZNF10, ZNF112, ZNF14, ZNF2, ZNF204P, ZNF248, ZNF382, ZNF420, ZNF512, ZNF564, ZNF569, ZNF571, ZNF573, ZNF594, ZNF607, ZNF709, ZNF780B, ZNF781, ZNF81, ZSCAN26, ZSWIM5 | >0.6 | >0.2 | 156 |
| turquoise | ABCG1, ABHD10, APLP1, ARHGEF9, ATP2A3, ATP6AP1, ATRNL1, BEX1, BEX4, BTBD3, C14orf132, CACNA1C, CACNA2D1, CADPS, CD200, CELF3, CHST9, CLCN4, CNTN1, COQ10A, CRMP1, CTNNA2, CXorf57, CYP2U1, CYYR1, DDX25, DLG2, DLG4, DOCK3, DPP6, DPYSL2, DUSP26, DYNC1I1, DZIP3, ELAVL4, ELOVL4, ENO2, EPM2AIP1, FAM171B, FGF14, FMN2, GABRB3, GAD2, GCNT1, GNAO1, GNAS, GNAZ, GNG2, GPR158, GPRASP1, GRK3, HADH, HCFC2, HMGCLL1, HSD17B14, IL15RA, IL4R, INA, INPP5F, JAZF1, KATNAL1, KCNB2, KCNJ6, KCNMA1, KIAA1324, KIF5C, KLHL42, KRT222, MAGI2, MAP6, MAPRE3, MBOAT2, MEIS2, MOB1B, NALCN, NAP1L2, NAP1L3, NAP1L5, NAPB, NBEA, NCALD, NEBL, NEUROD1, NEXMIF, NKX2-2, NLGN1, NMNAT2, NOL4, NRCAM, NXPE3, OTULINL, OXCT1, PAX6, PCLO, PCSK1, PDZD2, PELI3, PFKM, PFN2, PIAS1, PLCB4, PLCXD3, PNMA2, PPM1E, PPM1L, PPP2R2C, PTPRN, RAB39B, RAB3C, RAB9B, RCAN2, RCAN3, RGS7, RIMBP2, RIMS2, ROBO1, ROBO2, RPS6KA6, RTL5, RTN1, RUNDC3A, SALL2, SAMD3, SCG3, SCGN, SEMA5A, SH3GL2, SLC16A9, SLC22A17, SLC25A12, SLC30A4, SLC4A8, SLC7A8, ST18, STXBP1, STXBP5L, SV2A, SVOP, SYN1, SYP, SYT13, SYT14, SYT4, SYT7, TERF2IP, TMEM196, TMEM63C, TMOD2, TPD52, TSPAN2, TSPAN7, TSPYL5, UCHL1, UNC13A, UNC80, WDR7, WFS1 | >0.8 | >0.2 | 158 |

Supplementary Table 2 hub genes of turquoise module and purple module

Supplementary Table 3 CMap analysis results in ND-T2D

| Rank | Score | Name | MOA | Target |
| --- | --- | --- | --- | --- |
| 8550 | -98.45 | ISOX | HDAC inhibitor | HDAC6 |
| 8549 | -98.24 | panobinostat | HDAC inhibitor | HDAC1, HDAC2, HDAC3, HDAC4, HDAC6, HDAC7, HDAC8, HDAC9 |
| 8545 | -97.16 | belinostat | HDAC inhibitor | HDAC1, HDAC2, HDAC3, HDAC4, HDAC5, HDAC6, HDAC7, HDAC8, HDAC9 |
| 8542 | -96.34 | THM-I-94 | HDAC inhibitor | HDAC1, HDAC10, HDAC2, HDAC3, HDAC6, HDAC8 |
| 8541 | -96.19 | AS-703026 | MEK inhibitor | MAP2K1, MAP2K2 |
| 8540 | -96.17 | XMD-1150 | Leucine rich repeat kinase inhibitor | LRRK2 |
| 8538 | -95.5 | scriptaid | HDAC inhibitor | HDAC1, HDAC2, HDAC3, HDAC4, HDAC5, HDAC6, HDAC7, HDAC8, HDAC9 |
| 8529 | -92.15 | vorinostat | HDAC inhibitor | HDAC1, HDAC2, HDAC3, HDAC6, HDAC8, HDAC10, HDAC11, HDAC5, HDAC9 |
| 8528 | -92.11 | TG-101348 | FLT3 inhibitor, JAK inhibitor | JAK2, FLT3, BRD4, JAK1, JAK3, RET, TYK2 |
| 8525 | -91 | trichostatin-a | HDAC inhibitor, CDK activator, ID1 inhibitor | HDAC7, HDAC8, HDAC1, HDAC10, HDAC2, HDAC3, HDAC4, HDAC5, HDAC6, HDAC9 |
| 8521 | -88.97 | HC-toxin | HDAC inhibitor | HDAC1 |
| 8516 | -86.39 | NCH-51 | HDAC inhibitor | HDAC1, HDAC10, HDAC11, HDAC2, HDAC3, HDAC4, HDAC5, HDAC6, HDAC7, HDAC8, HDAC9 |
| 8514 | -84.92 | dacinostat | HDAC inhibitor | HDAC1, HDAC2, HDAC3, HDAC4, HDAC5, HDAC6, HDAC7, HDAC8, HDAC9 |
| 8513 | -84.91 | norethindrone | Progesterone receptor agonist, Progestogen hormone | PGR |
| 8504 | -83.62 | neratinib | EGFR inhibitor | EGFR, ERBB2, ERBB4, KDR |
| 8501 | -83.11 | prometon | Photosynthesis inhibitor |  |
| 8500 | -82.75 | LY-303511 | Casein kinase inhibitor, MTOR inhibitor, PI3K inhibitor | CSNK2A1, CSNK2A2, CSNK2B, MTOR |
| 8494 | -81.41 | apicidin | HDAC inhibitor | HDAC1, HDAC10, HDAC11, HDAC2, HDAC3, HDAC4, HDAC5, HDAC6, HDAC7, HDAC8, HDAC9 |
| 8489 | -80.86 | pyroxamide | HDAC inhibitor | HDAC1 |
| 8488 | -80.57 | KU-0060648 | DNA dependent protein kinase inhibitor, PI3K inhibitor | PIK3CA, PIK3CB, PIK3CD, PIK3CG, PRKDC |

Supplementary Table 4 CMap analysis results in IGT-T2D

| Rank | Score | Name | MOA | Target |
| --- | --- | --- | --- | --- |
| 8551 | -96.92 | PD-184352 | MEK inhibitor | MAP2K1, MAP2K2, MAP3K1, MAP3K2 |
| 8541 | -88.39 | AS-703026 | MEK inhibitor | MAP2K1, MAP2K2 |
| 8540 | -88.3 | itopride | Dopamine receptor antagonist | DRD2, ACHE, CHRM3 |
| 8537 | -84.12 | TG-101348 | FLT3 inhibitor, JAK inhibitor | JAK2, FLT3, BRD4, JAK1, JAK3, RET, TYK2 |
| 8533 | -78.4 | ascorbyl-palmitate | antioxidant |  |
| 8532 | -77.69 | AS-605240 | PI3K inhibitor | MAOB, PIK3CA, PIK3CB, PIK3CD, PIK3CG |
| 8528 | -73.58 | scandenin | Plant compound with antimicrobial activity |  |
| 8520 | -70.32 | SB-239063 | p38 MAPK inhibitor | MAPK11, MAPK14, PTGS2, TNF |
| 8517 | -69.39 | CL-218872 | GABA receptor agonist | GABRA1, GABRA2, GABRA3, GABRA5, GABRB2, GABRG2 |
| 8515 | -68.5 | baeomycesic-acid | Lipoxygenase inhibitor |  |
| 8505 | -64.15 | YM-298198 | Glutamate receptor antagonist | GRM1 |
| 8502 | -63.08 | MEK1-2-inhibitor | MEK inhibitor | MAP2K1, MAP2K2 |
| 8500 | -61.88 | LY-303511 | Casein kinase inhibitor, MTOR inhibitor, PI3K inhibitor | CSNK2A1, CSNK2A2, CSNK2B, MTOR |
| 8494 | -59.6 | fostamatinib | SYK inhibitor | SYK, FLT3, RET |
| 8493 | -59.17 | selumetinib | MEK inhibitor | MAP2K1, MAP2K2 |
| 8492 | -58.34 | UNC-0321 | Histone lysine methyltransferase inhibitor | EHMT2 |
| 8486 | -54.33 | oxybutynin | Acetylcholine receptor antagonist | CHRM3, CHRM1, CHRM2, CHRM4 |
| 8484 | -53.93 | AZ-628 | RAF inhibitor | BRAF, RAF1 |
| 8481 | -52.24 | SB-221284 | Serotonin receptor antagonist | HTR2B, HTR2C, HTR2A |
| 8480 | -52.02 | methyllycaconitine | Acetylcholine receptor antagonist | CHRNA7 |

Supplementary Table 5 Properties of Effective Drug-like Compounds of GLQMW

| number | compound | structure | OB（%） | Caco-2 | DL | herb |
| --- | --- | --- | --- | --- | --- | --- |
| QM1 | dianoside A_qt | 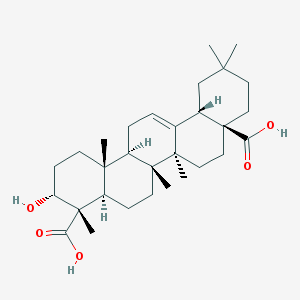 | 37.17 | 0.01 | 0.73 | Dianthi Herba |
| THF1 | Schottenol | 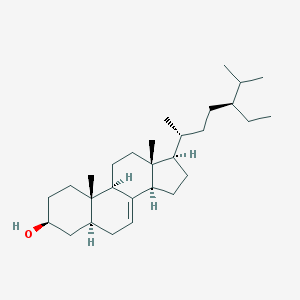 | 37.42 | 1.33 | 0.75 | Trichosanthis Radix |
| THF2 | Spinasterol | 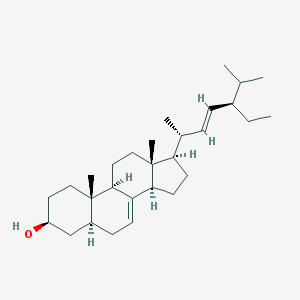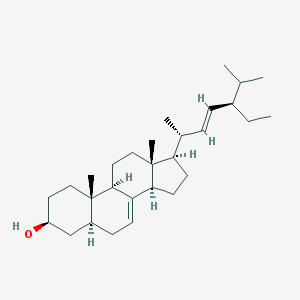 | 42.98 | 1.44 | 0.76 | Trichosanthis Radix |
| FL1 | (2R)-2-[(3S,5R,10S,13R,14R,16R,17R)-3,16-dihydroxy-4,4,10,13,14-pentamethyl-2,3,5,6,12,15,16,17-octahydro-1H-cyclopenta[a]phenanthren-17-yl]-5-isopropyl-hex-5-enoic acid | 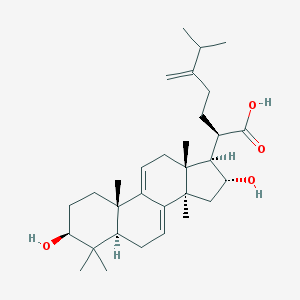 | 31.07 | 0.05 | 0.82 | Poria Cocos (Schw.) Wolf. |
| FL2 | hederagenin | 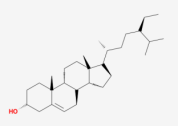 | 36.91 | 1.32 | 0.75 | Poria Cocos (Schw.) Wolf. |
| FL3 | pachymic acid | 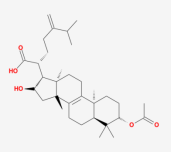 | 33.63 | 0.1 | 0.81 | Poria Cocos (Schw.) Wolf. |
| FL4 | Poricoic acid A | 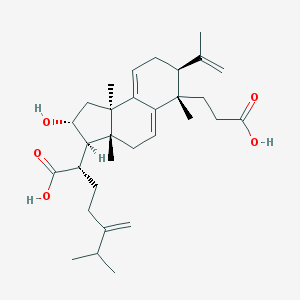 | 30.61 | -0.14 | 0.76 | Poria Cocos (Schw.) Wolf. |
| FL5 | Poricoic acid B | 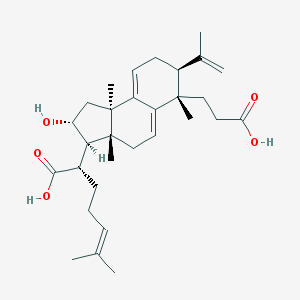 | 30.52 | -0.08 | 0.75 | Poria Cocos (Schw.) Wolf. |
| FL6 | poricoic acid C | 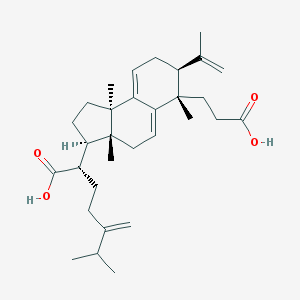 | 38.15 | 0.32 | 0.75 | Poria Cocos (Schw.) Wolf. |
| FL7 | trametenolic acid | 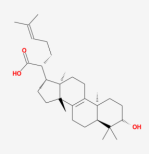 | 38.71 | 0.52 | 0.8 | Poria Cocos (Schw.) Wolf. |
| FL8 | (2R)-2-[(3S,5R,10S,13R,14R,16R,17R)-3,16-dihydroxy-4,4,10,13,14-pentamethyl-2,3,5,6,12,15,16,17-octahydro-1H-cyclopenta[a]phenanthren-17-yl]-6-methylhept-5-enoic acid | 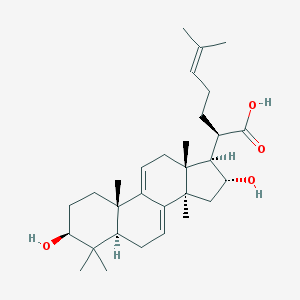 | 30.93 | 0.01 | 0.81 | Poria Cocos (Schw.) Wolf. |
| FL9 | (2R)-2-[(5R,10S,13R,14R,16R,17R)-16-hydroxy-3-keto-4,4,10,13,14-pentamethyl-1,2,5,6,12,15,16,17-octahydrocyclopenta[a]phenanthren-17-yl]-5-isopropyl-hex-5-enoic acid | 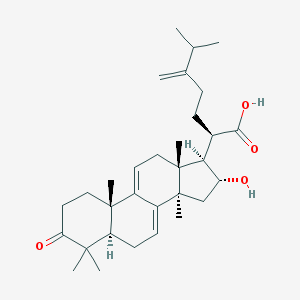 | 38.26 | 0.12 | 0.82 | Poria Cocos (Schw.) Wolf. |
| FL10 | 3beta-Hydroxy-24-methylene-8-lanostene-21-oic acid | 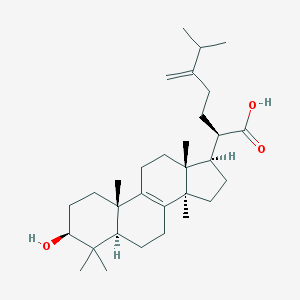 | 38.7 | 0.61 | 0.81 | Poria Cocos (Schw.) Wolf. |
| FL11 | 7,9(11)-dehydropachymic acid | 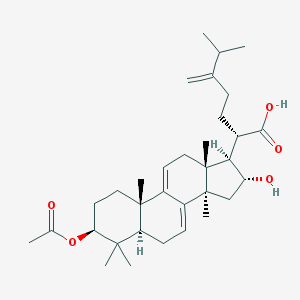 | 35.11 | 0.03 | 0.81 | Poria Cocos (Schw.) Wolf. |
| FL12 | Cerevisterol | 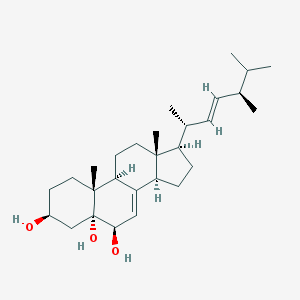 | 37.96 | 0.28 | 0.77 | Poria Cocos (Schw.) Wolf. |
| FL13 | dehydroeburicoic acid | 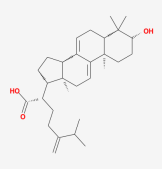 | 44.17 | 0.38 | 0.83 | Poria Cocos (Schw.) Wolf. |
| FL14 | ergosta-7,22E-dien-3beta-ol | 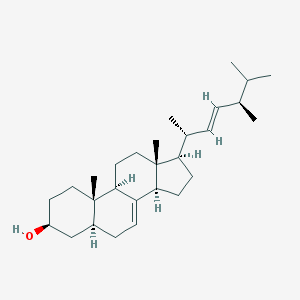 | 43.51 | 1.32 | 0.72 | Poria Cocos (Schw.) Wolf. |
| FL15 | Ergosterol peroxide | 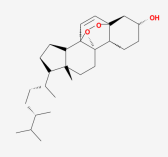 | 40.36 | 0.84 | 0.81 | Poria Cocos (Schw.) Wolf. |
| FZ1 | Neokadsuranic acid B | 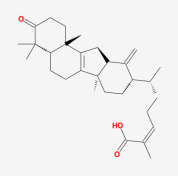 | 43.1 | 0.69 | 0.85 | Aconiti Lateralis Radix Praeparata |
| FZ2 | Carnosifloside I_qt | 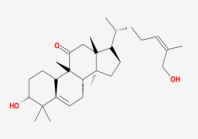 | 38.16 | 0.28 | 0.8 | Aconiti Lateralis Radix Praeparata |
| FZ3 | sitosterol | 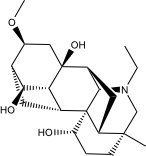 | 36.91 | 1.32 | 0.75 | Aconiti Lateralis Radix Praeparata |
| FZ4 | karakoline | 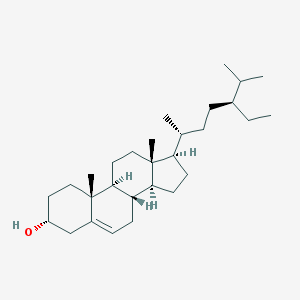 | 51.73 | 0.32 | 0.73 | Aconiti Lateralis Radix Praeparata |
| FZ5 | isotalatizidine | 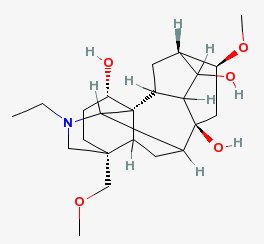 | 50.82 | -0.11 | 0.73 | Aconiti Lateralis Radix Praeparata |
| FZ6 | 6-Demethyldesoline | 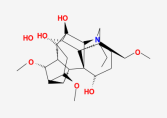 | 51.87 | -0.26 | 0.66 | Aconiti Lateralis Radix Praeparata |
| FZ7 | benzoylnapelline | 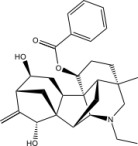 | 34.06 | 0.19 | 0.53 | Aconiti Lateralis Radix Praeparata |
| FZ8 | Deltoin | 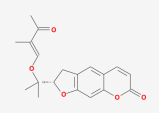 | 46.69 | 0.55 | 0.37 | Aconiti Lateralis Radix Praeparata |
| FZ9 | Karanjin | 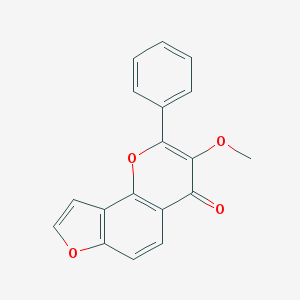 | 69.56 | 1.22 | 0.34 | Aconiti Lateralis Radix Praeparata |
| FZ10 | Deoxyandrographolide | 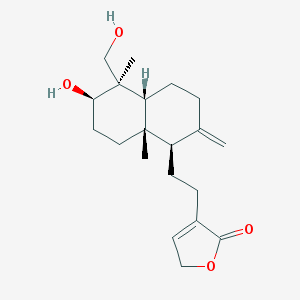 | 56.3 | 0.18 | 0.31 | Aconiti Lateralis Radix Praeparata |
| FZ11 | Delphin_qt | 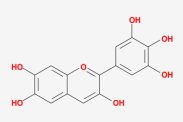 | 57.76 | 0.12 | 0.28 | Aconiti Lateralis Radix Praeparata |
| FZ12 | hypaconitine | 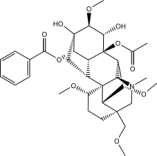 | 31.39 | -0.34 | 0.26 | Aconiti Lateralis Radix Praeparata |
| FZ13 | ignavine | 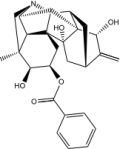 | 84.08 | -0.07 | 0.25 | Aconiti Lateralis Radix Praeparata |
| FZ14 | deoxyaconitine | 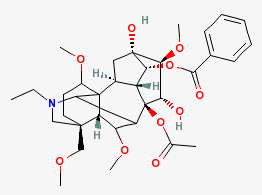 | 30.96 | -0.23 | 0.24 | Aconiti Lateralis Radix Praeparata |
| FZ15 | (R)-Norcoclaurine | 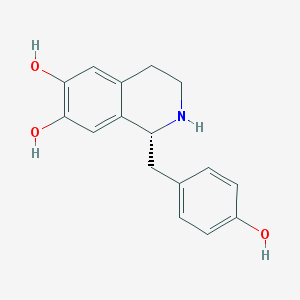 | 82.54 | 0.63 | 0.21 | Aconiti Lateralis Radix Praeparata |
| FZ16 | 11,14-eicosadienoic acid | 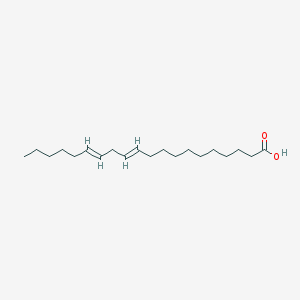 | 39.99 | 1.22 | 0.2 | Aconiti Lateralis Radix Praeparata |
| SY1 | piperlonguminine | 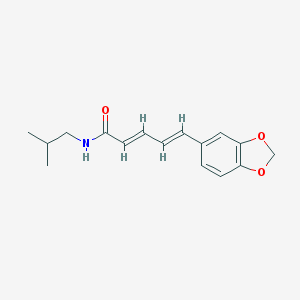 | 30.71 | 0.95 | 0.18 | Rhizoma Dioscoreae |
| SY2 | Methylcimicifugoside_qt | 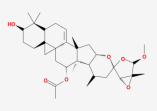 | 31.69 | 0.21 | 0.24 | Rhizoma Dioscoreae |
| SY3 | (-)-taxifolin | 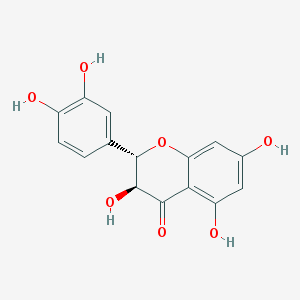 | 60.51 | -0.24 | 0.27 | Rhizoma Dioscoreae |
| SY4 | hancinol | 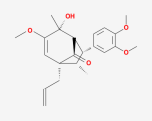 | 64.01 | 0.53 | 0.37 | Rhizoma Dioscoreae |
| SY5 | Kadsurenone | 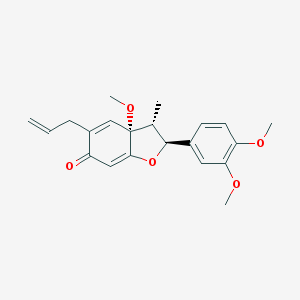 | 54.72 | 0.82 | 0.38 | Rhizoma Dioscoreae |
| SY6 | Denudatin B | 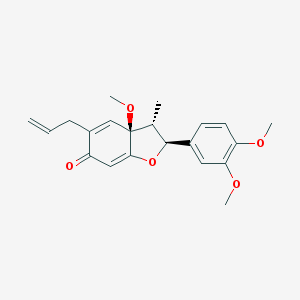 | 61.47 | 0.9 | 0.38 | Rhizoma Dioscoreae |
| SY7 | hancinone C | 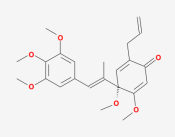 | 59.05 | 0.74 | 0.39 | Rhizoma Dioscoreae |
| SY8 | Doradexanthin | 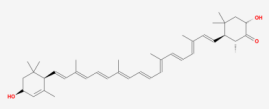 | 38.16 | 0.52 | 0.54 | Rhizoma Dioscoreae |
| SY9 | CLR | 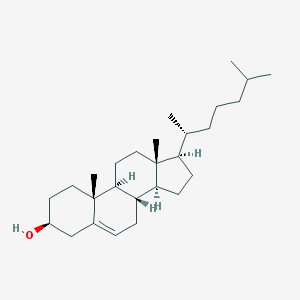 | 37.87 | 1.43 | 0.68 | Rhizoma Dioscoreae |
| SY10 | campesterol | 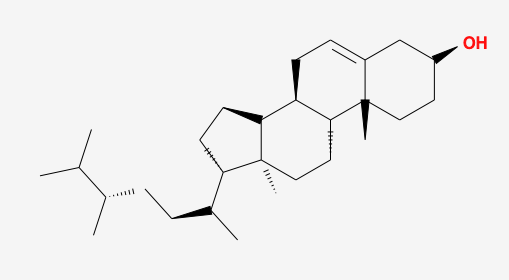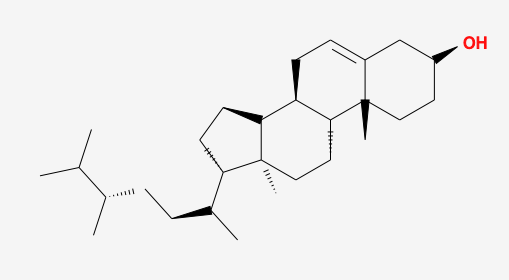 | 37.58 | 1.34 | 0.71 | Rhizoma Dioscoreae |
| SY11 | 24-Methylcholest-5-enyl-3belta-O-glucopyranoside_qt | 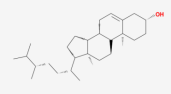 | 37.58 | 1.33 | 0.72 | Rhizoma Dioscoreae |
| SY12 | Stigmasterol | 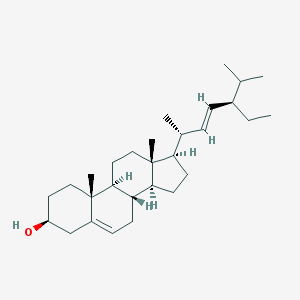 | 43.83 | 1.44 | 0.76 | Rhizoma Dioscoreae |
| SY13 | Isofucosterol | 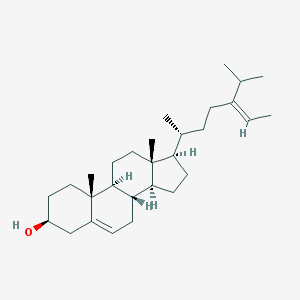 | 43.78 | 1.36 | 0.76 | Rhizoma Dioscoreae |
| SY14 | AIDS180907 | 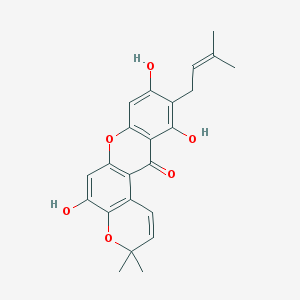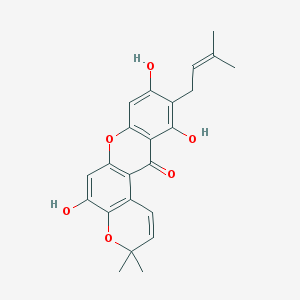 | 45.33 | 0.73 | 0.77 | Rhizoma Dioscoreae |
| SY15 | diosgenin | 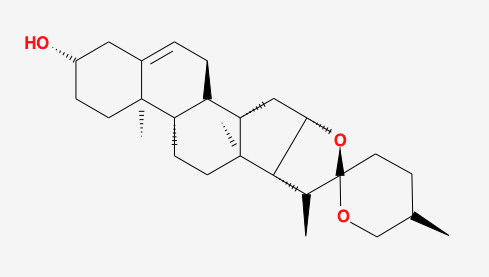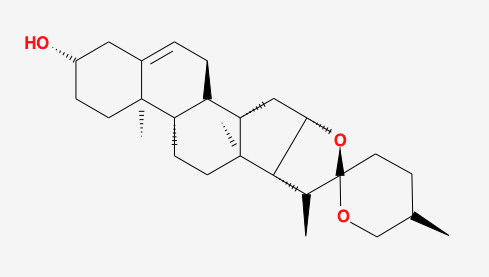 | 80.88 | 0.82 | 0.81 | Rhizoma Dioscoreae |
| SY16 | Dioscoreside C_qt | 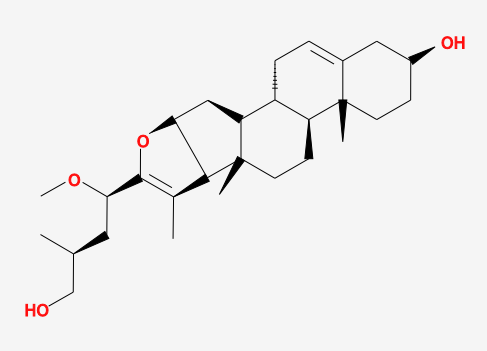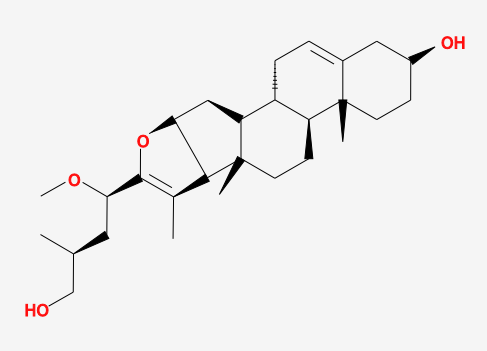 | 36.38 | 0.39 | 0.87 | Rhizoma Dioscoreae |

Supplementary Table 6 Binding energy between compound and protein

| **Target protein** | **Docking (binding energy) (kcal/mol)** | | |
| --- | --- | --- | --- |
|  | **(-)-taxifolin** | **dianosideA_qt** | **Spinasterol** |
| C1S |  | -8.4 | -9.2 |
| HSD11B | -7.5 | -8.8 | -8.5 |
| KIT |  | -8.6 | -9.2 |
| MET | -7.1 |  |  |
| PLAT | -7.6 |  |  |
| RBP4 | -6.2 | -7.5 |  |
